# Phytochemical Profiling of Mint and Basil Extracts and Their Association with Wastewater Biofilm-Associated Structures

**DOI:** 10.3390/molecules31213836

**Published:** 2026-10-23

**Authors:** Bidisha Sengupta, Olabisi Ogunlewe, Nkeiruka Angela Aziekwu, Ken Fiffick, Robert Friedfeld, Cephus Bess-Grunewald, Perla Tovar, Philip Baker, Thanh Tien Dao, Kylie Robertson, Michael Janusa, Paul Kim

**Affiliations:** 1 Department of Chemistry and Biochemistry, Stephen F. Austin State University, 1936 North Street, Nacogdoches, TX 75965, USA; ogunleweoo@jacks.sfasu.edu (O.O.); aziekwuna@jacks.sfasu.edu (N.A.A.); fiffickkm@jacks.sfasu.edu (K.F.); bessgruner@jacks.sfasu.edu (C.B.-G.); tovarpm@jacks.sfasu.edu (P.T.); bakerpa1@jacks.sfasu.edu (P.B.); daott@jacks.sfasu.edu (T.T.D.); robertsoka2@jacks.sfasu.edu (K.R.);; 2 Department of Engineering and Construction Sciences, Stephen F. Austin State University, 1936 North Street, Nacogdoches, TX 75965, USA; rfriedfeld@sfasu.edu; 3 Department of Biology, Grambling State University, 403 Main Street, Grambling, LA 71245, USA; kimp@gram.edu

**Keywords:** wastewater reuse, phytochemicals, luteolin, kaempferol, UPLC–HRMS/MS, scanning electron microscopy

## Abstract

Microbial biofilms in treated wastewater are a concern for water reuse because microorganisms can remain associated with surfaces after treatment. This study examined the flavonoid composition of methanolic extracts of peppermint (*Mentha × piperita*), spearmint (*Mentha spicata*), chocolate mint (*Mentha × piperita* f. *citrata* ‘Chocolate’), and basil (*Ocimum basilicum*) and investigated their association with Bacillus thuringiensis biofilms formed on silicon wafers in treated wastewater supplemented with Luria–Bertani (LB) broth. The final concentration of each plant extract was 0.3% (*v*/*v*). Ultra-performance liquid chromatography coupled with high-resolution tandem mass spectrometry (UPLC–HRMS/MS) was used to identify and quantify selected flavonoids. Scanning electron microscopy (SEM) and energy-dispersive X-ray spectroscopy (EDX) were used to examine surface morphology, bacterial-cell dimensions, and elemental composition. The extracts showed distinct phytochemical profiles. Luteolin-7-O-rutinoside was the major quantified flavonoid in the three mint extracts, whereas basil contained predominantly kaempferol-3-O-rutinoside and kaempferol-3-O-glucoside. SEM images showed differences in surface-associated structures among untreated, methanol-control, and plant-extract-treated samples. The longitudinal dimensions of selected dispersed bacterial cells also varied among the treatments. Thickness measurements were obtained for the untreated and methanol-control samples, while scattered, poorly defined structures in representative plant-extract-treated samples did not permit reliable thickness measurements. EDX analysis showed variation in the relative elemental composition of selected surface regions, with differences observed among treatments and between the two wastewater conditions. These findings provide a chemical and surface-characterization profile of the tested plant extracts and their association with B. thuringiensis biofilm-associated structures.

## 1. Introduction

The increasing demand for freshwater due to increase in population, industrialization, agriculture, and climate change have led to the need for alternative water resources [1,2]. Treated municipal wastewater has emerged as an essential element in sustainable water management due to its potential as an additional source for irrigation in agriculture and landscapes, industry use, and aquifer recharge. Despite the improvements that wastewater treatment processes offer in terms of water quality through decreasing concentrations of suspended solids, organic compounds, nutrients, and microorganisms, treated wastewater still retains the ability to allow for the development of microbial attachment and biofilm formation on submerged surfaces. Control of biofilm formation in reclaimed water systems is therefore a critical issue for further sustainable water reuse [3,4,5].

Biofilms are organized microbial communities encased in an extracellular polymeric substance (EPS) matrix that mainly comprises polysaccharides, proteins, lipids, and extracellular DNA [3,6,7]. The EPS matrix supports attachment to surfaces, retention of nutrients, and protection of the encapsulated microorganisms against environmental stress and chemical treatments. This leads to significantly increased resistance of biofilm-embedded microorganisms to antimicrobials when compared with their planktonic forms, making it difficult to remove them. The formation of biofilms can cause problems in wastewater treatment plants and distribution systems by leading to membrane fouling, blockage of pipelines, degradation of water quality, and increased expenses [5]. Thus, the development of environmentally friendly techniques for inhibition of microbial attachment and biofilm formation is highly relevant.

Our laboratory has been investigating microbial biofilms in environmental and biological systems using spectroscopic, microscopic, and biochemical approaches for several years [7,8,9]. In our previous investigation, we used *Bacillus thuringiensis* as the model organism for testing biofilm formation in chlorine- and sulfur dioxide- treated municipal wastewater samples collected from wastewater treatment facilities in Deep East Texas [10]. This highlights the need for sustainable approaches that can modulate biofilm development without relying on additional chemical disinfectants. Plant-derived phytochemicals can affect bacterial attachment, EPS production, quorum sensing, and biofilm maturation [11].

According to the recent studies, flavonoids and other polyphenols were able to affect bacteria adhesion, cell-to-cell communication, EPS synthesis, oxidative response and biofilm maturation but not via one antimicrobial function [12,13,14,15,16]. Peppermint (*Mentha × piperita*), spearmint (*Mentha spicata*), chocolate mint and basil (*Ocimum basilicum*) belong to the Lamiaceae family and are known for the high content of phenolic and flavonoid compounds and their abilities to inhibit bacteria growth and biofilm development [14,17,18,19]. Nevertheless, the phytochemical content of the plants depends on many factors such as species, cultivar, growing conditions and method of extraction. Thus, in order to establish relationships between chemical and biological properties of the plants under investigation, their chemical profile should be characterized first.

In addition, UPLC-HRMS MS (ultra-performance liquid chromatography coupled with High-resolution mass spectrometry) allows for accurate identification of flavonoids and other secondary metabolites in the plants. Thus, the aim of the current study was to study the impact of methanolic extracts of peppermint, spearmint, chocolate mint, and basil on Bacillus thuringiensis biofilms grown in chlorine and sulfur dioxide disinfected municipal wastewaters. UPLC-HRMS MS was utilized for the determination of the major phytochemical components in each of the extracts, whereas scanning electron microscopy and energy dispersive X-ray spectroscopy were used to analyze the alterations in biofilm morphology and chemical composition induced by different treatments on silicon substrates. This study builds on our previous study of biofilms in municipal wastewater from the Nacogdoches Wastewater Treatment Plant (NWWTP) [10], adding new data on the interaction of biofilms with flavonoid-containing plant extracts.

## 2. Materials and Methods

### 2.1. Plant Materials (Taxonomic Identification) and Preparation of Methanolic Extracts

**Scheme 1:**
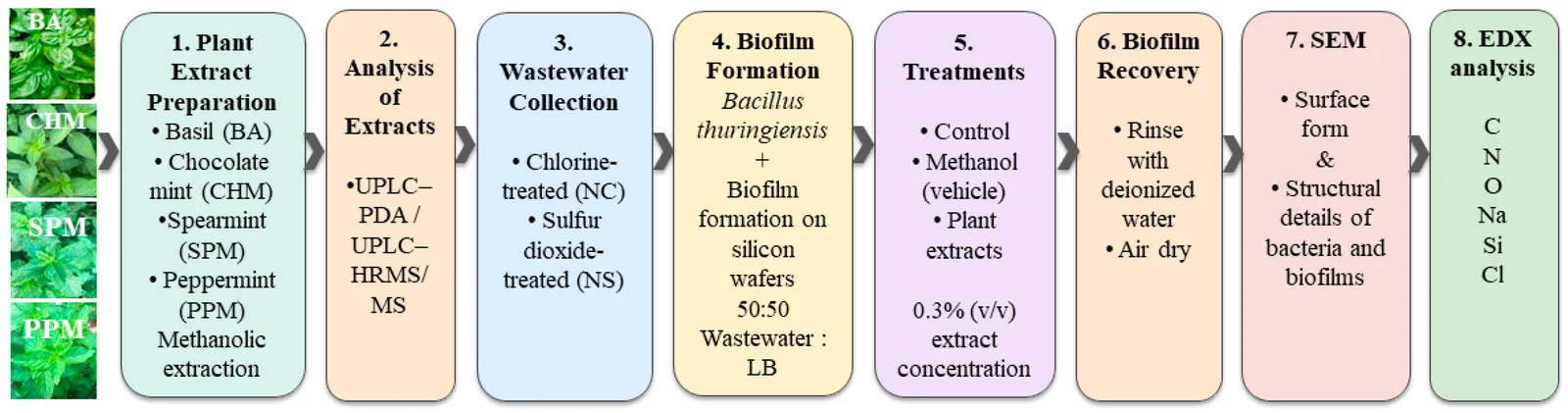
Experimental workflow used for biofilm cultivation on silicon wafers, treatment with flavonoid-rich plant extracts, and SEM/EDX characterization.

Fresh leaves of sweet basil (*Ocimum basilicum* L.), spearmint (*Mentha spicata* L.), peppermint (*Mentha × piperita* L.), and chocolate mint (*Mentha × piperita* f. *citrata* ‘Chocolate’) were collected from a local garden in Nacogdoches, Texas. Plant identification was based on the observed morphological characteristics of the collected leaves and comparison with reference descriptions and images from the Missouri Botanical Garden Plant Finder [20]. The taxonomic identities were further cross-referenced with the NCBI Taxonomy database [21]: *O. basilicum* (NCBI Taxon ID 39350), *M. spicata* (NCBI Taxon ID 29719), and *M. × piperita* (NCBI Taxon ID 34256). Chocolate mint was treated as the horticultural form *M. × piperita* f. *citrata* ‘Chocolate’; its NCBI taxonomic reference corresponds to the *M. × piperita* species node (Taxon ID 34256). Photographs of the plant materials used in the study are provided in Scheme 1 and the graphical abstract.

Methanolic extraction was adapted from our previously reported procedure for the extraction of plant phytochemicals [22]. Fresh leaves were thoroughly washed with distilled water and air-dried before extraction. Spearmint (1.2189 g) and peppermint (1.2173 g) leaves were each homogenized in 40 mL methanol, basil (2.646 g) leaves were homogenized in 60 mL methanol, and chocolate mint (0.5385 g) leaves were homogenized in 29 mL methanol. The corresponding starting leaf-to-solvent ratios were 0.03047, 0.03043, 0.04410, and 0.01857 g/mL for spearmint, peppermint, basil, and chocolate mint, respectively. These ratios describe the initial extraction loading; they do not represent the concentrations of individual phytochemicals in the resulting extracts.

The homogenized samples were stirred in methanol for 72 h at 4 °C and then centrifuged at 20,000× *g* for 30 min at 20 °C using a Beckman Coulter ultracentrifuge (Beckman Coulter Life Sciences, Indianapolis, IN, USA). The supernatants were collected, filtered to remove residual particulate matter, and passed through 0.22 µm sterile syringe filters. The resulting filtered methanolic extracts, containing the methanol-soluble constituents recovered from the leaf material, were stored at −20 °C until analysis and use. Individual target phytochemicals were quantified by UPLC–HRMS/MS using the calibration procedure described in Section 2.4. Concentrations measured in the diluted analytical solutions were back-calculated using the corresponding batch-specific dilution factors to obtain the reported concentrations in the original filtered extracts. Methanol was used as the extraction solvent for all four plant materials and was therefore included as the vehicle control in the biofilm experiments. The experimental workflow is presented in Scheme 1.

### 2.2. Wastewater Collection

Treated wastewater samples were collected from the NWWTP (Nacogdoches, TX, USA) as described previously [10]. Two sampling locations were selected to represent the final stages of the treatment process: the chlorine (Cl_2_) contact chamber (NC) and the sulfur dioxide (SO_2_) contact chamber (NS) following dichlorination, as described above and in our previous study [10]. Approximately 1 L of treated wastewater was collected from each location in sterile polypropylene bottles, transported to the laboratory on ice, and stored at 4 °C until use. All experiments were performed within 24 h of sample collection to minimize changes in water chemistry and microbial composition.

### 2.3. Physicochemical Characterization of NWWTP Effluent

The physicochemical characteristics of the treated wastewater, including chemical and biochemical oxygen demand (COD and BOD), total suspended solids (TSS), dissolved oxygen (DO), pH, and other relevant parameters, were reported in our previous publication [10]. To provide additional context on routine wastewater treatment plant conditions, operational effluent records from the NWWTP for November and December 2024 are presented in Supplementary Table S1. These records summarize daily measurements of BOD, TSS, DO, ammonia nitrogen, and pH. The supplementary data complement the previously published characterization and provide additional information on the variability of treated effluent quality.

### 2.4. UPLC–High-Resolution Mass Spectrometry (HRMS)

The phytochemical compositions of methanolic (MET) extracts prepared from peppermint (PPM), spearmint (SPM), chocolate mint (CHM), and basil (BA) were characterized using ultra-performance liquid chromatography coupled with high-resolution mass spectrometry (HRMS) (UPLC–HRMS/MS). Analyses were performed on a Vanquish UPLC system coupled to a Q-Exactive Plus HRMS mass spectrometer (Thermo Fisher Scientific, Waltham, MA, USA). The HRMS mass spectrometer was operated in electrospray ionization (ESI) mode using full-scan MS with data-dependent tandem mass spectrometry (ddMS^2^). Compounds were identified by comparing retention times (RT), accurate mass measurements, and MS/MS fragmentation patterns with those of authentic reference standards.

**Scheme 2:**
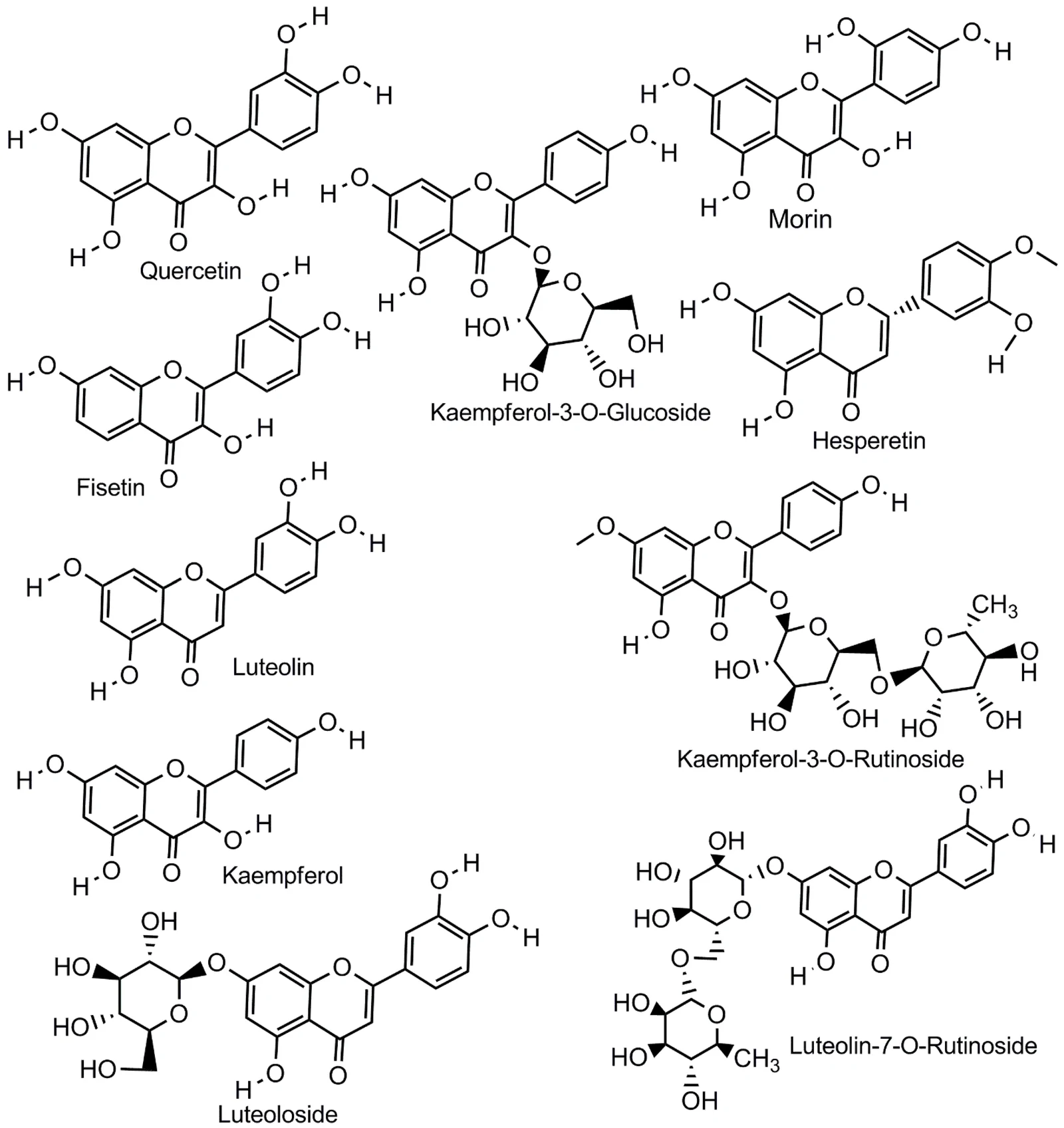
Chemical structures of flavonoids identified or evaluated in the mint and basil extracts, including quercetin, morin, fisetin, luteolin, kaempferol, hesperetin, luteoloside, luteolin-7-O-rutinoside, kaempferol-3-O-glucoside, and kaempferol-3-O-rutinoside.

Reference standards of kaempferol (KMP), kaempferol-3-*O*-β-D-glucoside (Kmp3G), kaempferol-3-*O*-rutinoside (Kmp3R), luteolin (Lut), luteoloside (Lutd), luteolin-7-rutinoside (Lut7R), quercetin (Quer), fisetin (Fis), hesperetin (Hes), quercetin-d_3_, fisetin-d_5_, and hesperetin-^13^C-d_3_ were obtained from Cayman Chemical (Ann Arbor, MI, USA), while morin was purchased from Sigma-Aldrich (St. Louis, MO, USA). Chemical structures of the reference standards are included in Scheme 2. UPLC-grade methanol and water, HPLC-grade acetic acid, HPLC-grade ammonium acetate (Fisher Chemical, Waltham, MA, USA), and ammonium fluoride (98% purity; Sigma-Aldrich, St. Louis, MO, USA) were used throughout the study.

Chromatographic separation was carried out using an Acclaim™ RSLC 120 C18 analytical column (100 × 2.1 mm, 2.2 μm particle size; Thermo Fisher Scientific) maintained at 40 °C. The mobile phase consisted of (A) water containing 10 mM ammonium acetate, 0.25 mM ammonium fluoride, and 0.1% acetic acid, and (B) methanol containing 10 mM ammonium acetate, 0.25 mM ammonium fluoride, and 0.1% acetic acid. Both plant extracts and calibration standards were prepared in a 50:50 (*v*/*v*) methanol-water solution prior to analysis. The UPLC gradient program is shown in Figure S1, whereas HRMS source settings and ddMS^2^ inclusion list are shown in Tables S2 and S3, respectively.

Quantitative analysis was performed using external calibration curves prepared from mixed flavonoid standards. A primary stock solution (1250 ng mL^−1^) was prepared and used to generate calibration standards ranging from 50–1000 ng mL^−1^. A separate secondary stock solution (50 ng mL^−1^) was also prepared and used to prepare calibration standards ranging from 5–25 ng mL^−1^. Calibration curves were generated by linear regression using 1/x weighting, without forcing the regression through the origin, based on analyte-to-internal-standard peak-area ratios. The low-quality control (LQC) was prepared from the secondary stock, whereas the medium-quality control (MQC) and high-quality control (HQC) were prepared from the 1250 ng mL^−1^ stock. Calibration parameters and coefficients of determination for each analytical batch are provided in Tables S4–S6.

Plant extracts were diluted prior to injection using batch-specific dilution factors to ensure that analyte responses fell within the validated calibration range. The dilution factors were 10-fold, 1000-fold, 100-fold, and 100,000-fold for Batches 1–4, respectively. The resulting analyte concentrations in the diluted analytical solutions fell within the respective 5–1000 ng mL^−1^ calibration ranges. Concentrations measured in the diluted analytical solutions were subsequently multiplied by the applicable dilution factor to obtain concentrations in the original filtered extracts. All calibration standards and plant extracts were spiked with 20 ng mL^−1^ isotopically labeled internal standards. All samples were analyzed in three analytical replicates, and mean concentrations and %CV were calculated using Thermo Scientific TraceFinder 5.1 (Thermo Fisher Scientific) and Microsoft Excel.

#### Isomer Differentiation by UPLC–HRMS/MS

For compounds with identical precursor masses, chromatographic retention behavior was used to distinguish peaks corresponding to individual standards. A modified chromatographic procedure was developed to separate structural isomers that could not be differentiated solely on the basis of accurate mass. This procedure was not used for quantitation of the analyzed plant extracts. Fisetin, luteolin, and kaempferol, having common molecular formula C_15_H_10_O_6_ and deprotonated molecular ion *m*/*z* 285.040, were analyzed as separate standards and in mixture under modified chromatographic conditions. Morin and quercetin, sharing the same molecular formula C_15_H_10_O_7_ and deprotonated molecular ion at *m*/*z* 301.035, were also analyzed in order to obtain their relative retention times. The procedure for the separation of isomers included the same column, flow rate, temperature, and generally the same gradient as described before, but two changes were introduced: the concentration of ammonium acetate in the mobile phase was decreased from 10 to 5 mM, and the proportion of the organic phase at 6 min of run time was changed from 86% to 85% B (Figure S2). These changes resulted in the slight change in retention times while keeping the relative order of elution for isomeric standards.

All samples were measured in three replicates. After the quantitation procedure, ddMS^2^ spectra were recorded in order to identify compounds based on comparison of fragmentation patterns with those of their respective reference standards. All procedures of instrument control, data collection, and quantification were conducted using Thermo Scientific TraceFinder 5.1, and mean concentration values and coefficient of variations (%CV) were calculated in Microsoft Excel.

### 2.5. Bacterial Culture and Biofilm Formation

Overnight cultivation of *Bacillus thuringiensis* (*Bt*) was performed at 37 °C with gentle shaking in Luria–Bertani (LB) broth. After 24 h, the bacterial culture reached an OD_600_ of approximately 0.9. The bacterial suspension was diluted using a 50:50 (*v*/*v*) mixture of treated wastewater and sterile LB broth to obtain a final OD_600_ of approximately 0.03, as described previously [10]. Silicon wafers were obtained from Nova Wafers (Argyle, TX, USA; Item No. A01-19996). The characteristics of silicon wafers are as follows: 1inch-diameter, p-type, boron-doped silicon, ⟨100⟩-oriented, single-side-polished with a resistivity of 1–10 Ω·cm and a thickness of 300 ± 25 µm. The wafers were gently rinsed with sterile deionized water and allowed to air-dry at room temperature individually in sterile 35 mm Petri dishes before use. Each wafer was then submerged in 4.0 mL of the diluted bacterial culture. The final concentration of the methanolic plant extracts was set at 0.3% (*v*/*v*) to maintain a low solvent concentration in the wastewater–LB medium. This choice was informed in part by our previous study, in which 0.5% (*v*/*v*) ethanol did not measurably alter the spectroscopic properties of human serum albumin under the conditions tested [22]. Methanol-only vehicle controls were included under both NC and NS conditions to assess the effect of methanol alone on biofilm development. The treatments included peppermint, spearmint, chocolate mint, and basil extracts, together with a methanol vehicle control and an untreated control. The wafers were incubated under static conditions at 37 °C for 24 h. Following incubation, the supernatant was aspirated, and the wafers were gently rinsed with sterile deionized water to remove loosely associated material. All experiments, including controls and treatments, were performed in two independent experimental runs, while selected experiments involving NC and NS were repeated in three independent runs.

### 2.6. Scanning Electron Microscopy (SEM)

Following incubation, the silicon wafers were gently rinsed with sterile deionized water and air dried at room temperature. The dried wafers were mounted on aluminum SEM stubs using carbon tape. No chemical fixation or conductive metal coating was applied. Surface morphology was examined using the SEM described in our previous wastewater study [10]. Images were acquired at an accelerating voltage of 20 kV, a working distance of approximately 15 mm, and a magnification of 4000× unless specified otherwise. Multiple fields of view were examined to document the surface-associated structures under each treatment condition.

### 2.7. Energy-Dispersive X-Ray Spectroscopy (EDX)

Elemental analysis was performed following SEM imaging using the integrated energy-dispersive X-ray spectroscopy (EDX) detector. Four regions of interest (ROIs) were selected from different locations on each silicon wafer for the bacteria-exposed samples, the bare wafer, and the abiotic controls. The abiotic controls consisted of wafers incubated in 50% (*v*/*v*) treated wastewater and LB medium containing 0.3% (*v*/*v*) chocolate mint extract in the absence of bacteria. Elemental weight percentages were obtained using the instrument software for carbon (C), nitrogen (N), oxygen (O), sodium (Na), silicon (Si), chlorine (Cl), and other detected elements. For each ROI, the composite elemental ratio (C + N + O + Na + Cl)/Si was calculated from the corresponding weight percentages. The mean and standard deviation of the four ROI-level ratios were calculated to describe spatial variability in surface composition. Because these ROIs represent spatial subsampling of a single wafer instead of independent biological replicates, the standard deviation reflects variation among sampled locations and not biological replicate variability. The ratios were treated as descriptive indicators of localized surface composition, not as direct measures of biofilm biomass, viability, or extracellular polymeric substance content, because the measured signals may include contributions from bacterial cells, extracellular material, plant extract residues, growth medium, wastewater constituents, and the silicon substrate.

For the separate EDX comparison associated with the SEM images used for biofilm thickness measurements, one ROI was selected from the dense biofilm area of each sample. The elemental composition of each selected ROI was used for the comparison presented in the corresponding EDX figure. This targeted EDX analysis complemented the biofilm thickness images presented in the main text by providing supporting information on the elemental composition of selected dense-biofilm regions. Together, the thickness images and corresponding EDX profiles provide a qualitative, complementary view of treatment-associated variations in biofilm structure and surface composition.

## 3. Results and Discussion

### 3.1. Phytochemical Characterization of Mint and Basil Extracts by UPLC–HRMS/MS

**Figure 1:**
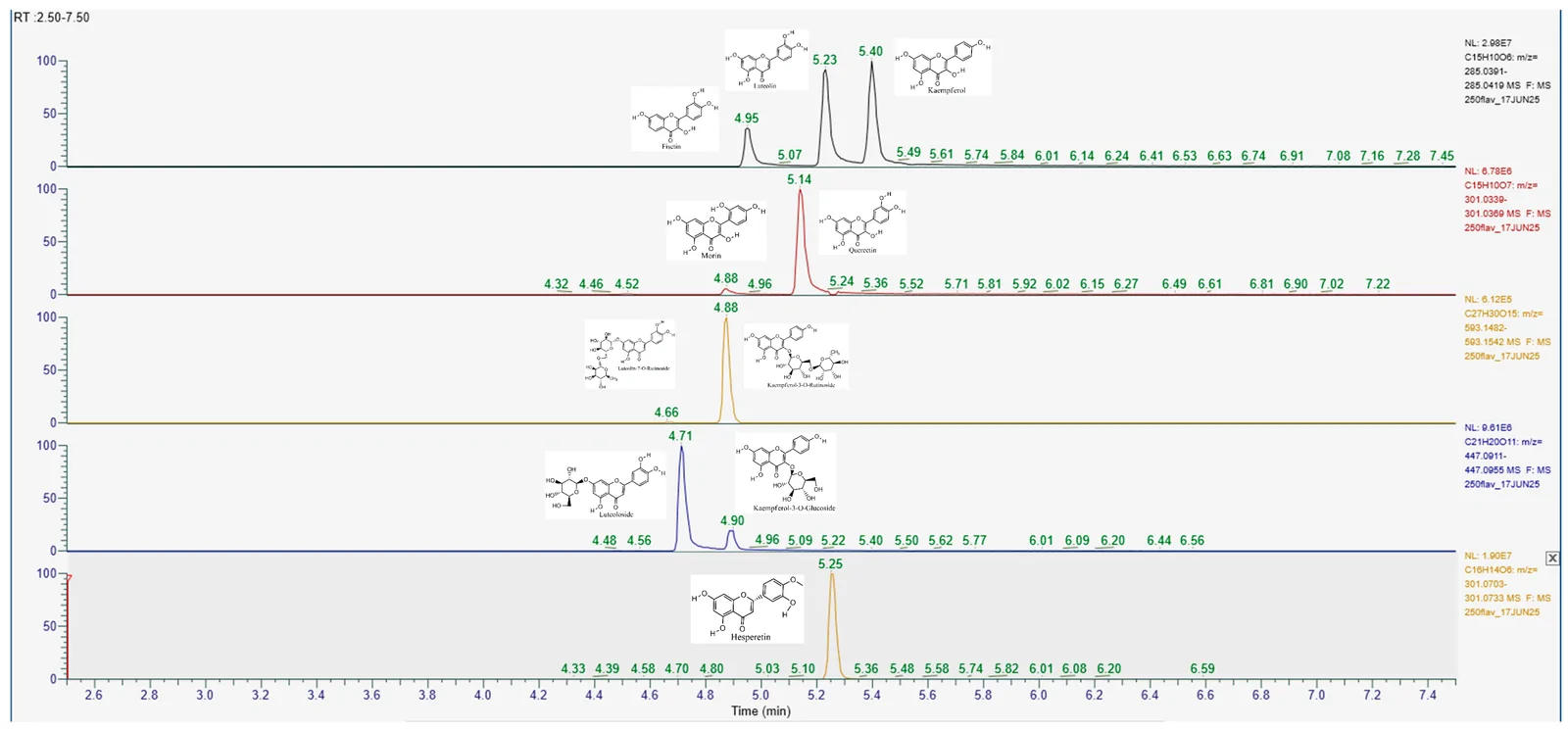
Chromatographic differentiation of isomeric flavonoid standards using the modified UPLC method. Extracted ion chromatograms of authentic flavonoid standards demonstrating chromatographic resolution of compounds sharing identical precursor masses.

Several of the flavonoids (included in Scheme 2) that have been used in the study are structural isomers and hence cannot be distinctly separated through their accurate precursor masses. To solve this problem of analysis, a modified UPLC method was formulated for separation of the isomers as provided in Section 2.3. The three isomers, namely, fisetin, luteolin, and kaempferol possess the same molecular formula, C_15_H_10_O_6_ and yield the same deprotonated precursor ions of *m*/*z* 285.040. Through the modified UPLC procedure, the three isomers were separated based on the retention times of 4.95, 5.23, and 5.40 min respectively giving an elution order of fisetin, luteolin, and kaempferol (as is presented in Figure 1). It can thus be noted that luteolin is the second chromatographic peak amongst the *m*/*z* 285 isomers. Likewise, the two isomers, morin and quercetin (with the same molecular formula, C_15_H_10_O_7_), having the same precursor ion at *m*/*z* 301.035, were separated with retention times of 4.88 and 5.14 min respectively. Other pairs of isomers luteoloside and kaempferol-3-O-glucoside (with the same molecular formula, C_21_H_20_O_11_) and luteolin-7-O-rutinoside and kaempferol-3-O-rutinoside (with the same molecular formula, C_27_H_30_O_15_) were tested using their chromatographic retentions as is evident in Figure 1. These isomeric pairs share identical precursor masses and molecular formulas, and their assignments therefore relied on chromatographic retention behavior established using authentic standards instead of accurate precursor mass alone. Hesperetin was monitored separately using its accurate precursor mass at *m*/*z* 301.071. Table 1 presents the limit of detections (LOD), limit of quantifications (LOQ) and upper limit of quantifications (ULOQ). LOD, LOQ and ULOQ were determined by diluting standards until no peak formation was present, measuring stability via %CV across triplicate injections and observing the experimentally highest calibration point respectively.

Figure S3 shows a representative ddMS^2^ differentiation of luteolin and fisetin. Both compounds produced a deprotonated molecular ion at approximately *m*/*z* 285.040, consistent with their structural isomerism. Despite having the same precursor mass, luteolin and fisetin exhibited distinct product-ion profiles. Luteolin showed a prominent product ion at *m*/*z* 133.028, whereas fisetin exhibited a different fragmentation pattern, including prominent product ions at *m*/*z* 121.028 and 135.007. The distinct MS/MS profiles provided complementary evidence for differentiation of the two isomers in conjunction with their chromatographic retention times. The top three product ion qualifiers for all analytes in this study are provided in Table S7.

**Figure 2:**
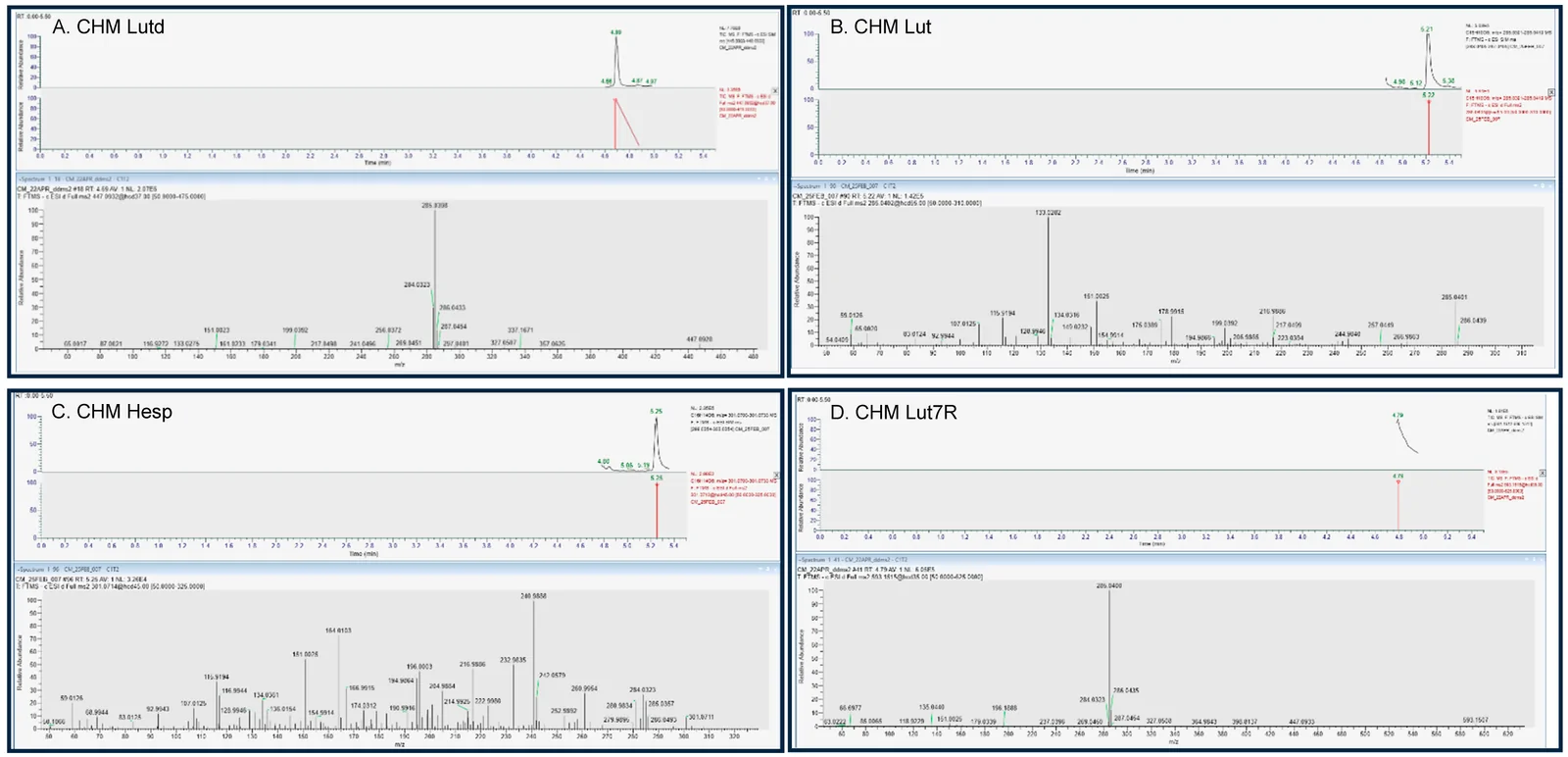
Typical UPLC–HRMS/MS using data-dependent MS/MS (ddMS^2^) chromatograms and corresponding MS/MS spectra of selected flavonoids in chocolate mint (CHM) extract. (**A**) Luteoloside (Lutd), (**B**) luteolin (Lut), (**C**) hesperetin (hesp) and (**D**) luteolin-7-O-rutinoside (Lut7R) detected in chocolate mint extract. Agreement in chromatographic retention times and characteristic precursor/product-ion patterns between the extract and corresponding authentic standards (Figure S4) supports the identification of these flavonoids in chocolate mint.

Figure S4 presents the typical UPLC–HRMS/MS using data-dependent MS/MS (ddMS^2^) chromatograms and corresponding MS/MS spectra of flavonoid standards: A Luteoloside (Lutd), B luteolin (Lut), C hesperetin (Hesp), and D luteolin-7-O-rutinoside (Lut7R). The retention times established using authentic standards were subsequently used for compound assignments in the mint extracts and representative UPLC–HRMS/MS/MS data are provided in Figure 2, Figures S5 and S6. Additional chromatographic features were observed in the plant extracts; however, their low signal intensity prevented reliable ddMS^2^ acquisition, and these features were therefore not assigned.

Figure 2 illustrates the application of the chromatographic and mass-spectrometric approach to the chocolate mint extract. For each selected compound, the chromatographic behavior of the extract was compared directly with that of the corresponding authentic standard, followed by comparison of the associated high-resolution MS/MS spectra. Lutd was found to produce peaks at the precursor mass *m*/*z* 447.093 with fragmentation giving the specific peak of the luteolin aglycone ion at approximately *m*/*z* 285.040 (Figure 2A and Figure S4A). Lut produced the peak at the precursor mass *m*/*z* 285.040 and specific fragments corresponding to the authentic luteolin standard (Figure 2B and Figure S4B). The peaks of Hesp were detected at its accurate precursor mass of *m*/*z* 301.071 with the fragmentation pattern corresponding to the authentic standard (Figure 2C and Figure S4C). Likewise, the peaks of Lut7R were detected at its accurate precursor mass *m*/*z* 593.151 with fragmentation to the specific peak of the luteolin aglycone at *m*/*z* 285, corresponding to the authentic standard (Figure 2D and Figure S4D). Thus, the agreement in chromatographic behavior and high-resolution MS/MS characteristics provided orthogonal evidence supporting the assignment of these four flavonoids in chocolate mint. For the basil extract, authentic standards of (A) kaempferol-3-O-glucoside (KMP3G), (B) kaempferol-3-O-rutinoside (KMP3R), and (C) luteolin (Lut) were analyzed separately to establish their chromatographic retention times and MS/MS fragmentation profiles (Figure S7). The corresponding chromatographic and MS/MS profiles observed in the basil extract are presented in Figure S8.

The quantitative analysis showed distinct differences in the levels and distribution of the targeted flavonoids among the four plant extracts (Table 2). Lut7R is the main quantified flavonoid in spearmint, peppermint, and chocolate mint, which have levels of 19.0, 21.0, and 21.0 mg/mL, respectively. The levels of luteoloside and luteolin were much lower in the three *Mentha* plants. On the other hand, hesperetin was found only in spearmint and chocolate mint, but not in peppermint. However, basil had a different pattern of distribution, where kaempferol-3-O-rutinoside and kaempferol-3-O-glucoside were the main quantified flavonoids, with luteolin at trace levels. The predominance of luteolin derivatives in the *Mentha* extracts and kaempferol glycosides in basil was consistent with previously reported phytochemical profiles of these plants [17,18,19]. These compositional differences provided the chemical context for interpreting treatment-dependent biofilm responses. However, the differences in phytochemical profiles should also be considered in the context of the plant-material-to-solvent loading used during extraction. The extraction loadings were 0.03047 g/mL for spearmint, 0.03043 g/mL for peppermint, 0.04410 g/mL for basil, and 0.01857 g/mL for chocolate mint. Thus, the extracts were not prepared from identical amounts of plant material per unit volume of methanol. Differences in extraction loading, together with plant-specific chemical composition, may have contributed to differences in the concentrations and relative abundance of the detected compounds.

### 3.2. Scanning Electron Microscopy and EDX Analysis

#### 3.2.1. Abiotic Controls

In order to establish a baseline for interpreting the SEM and EDX results, an unexposed silicon wafer and wafers exposed to the abiotic NC and NS wastewater in LB matrices containing 0.3% (*v*/*v*) chocolate mint (CHM) extract were examined in the absence of *Bacillus thuringiensis* (Figure S9). The abiotic NC and NS samples showed surface-associated features that differed from the relatively uniform surface of the unexposed wafer. These observations indicate that material from the treatment matrix remained associated with the wafer surface after sample preparation, even without bacteria.

EDX analysis supported this observation. The unexposed wafer contained approximately 99.0 wt% silicon, whereas the abiotic NC and NS samples contained 76.5 and 79.9 wt% silicon, respectively. Carbon and nitrogen were also detected in both abiotic samples. The NC sample contained 16.3 wt% carbon and 2.2 wt% nitrogen, while the NS sample contained 14.0 wt% carbon and 1.9 wt% nitrogen. Sodium and chlorine were also detected in the treated samples. These findings show that carbon and nitrogen signals cannot be attributed exclusively to bacterial cells or extracellular polymeric substances. Contributions from the wastewater in LB medium and plant extract must also be considered when interpreting the EDX results from the *Bt* containing samples.

#### 3.2.2. SEM Morphology, Dispersed Bacterial-Cell Dimensions, and Four-ROI Elemental Analysis

**Figure 3:**
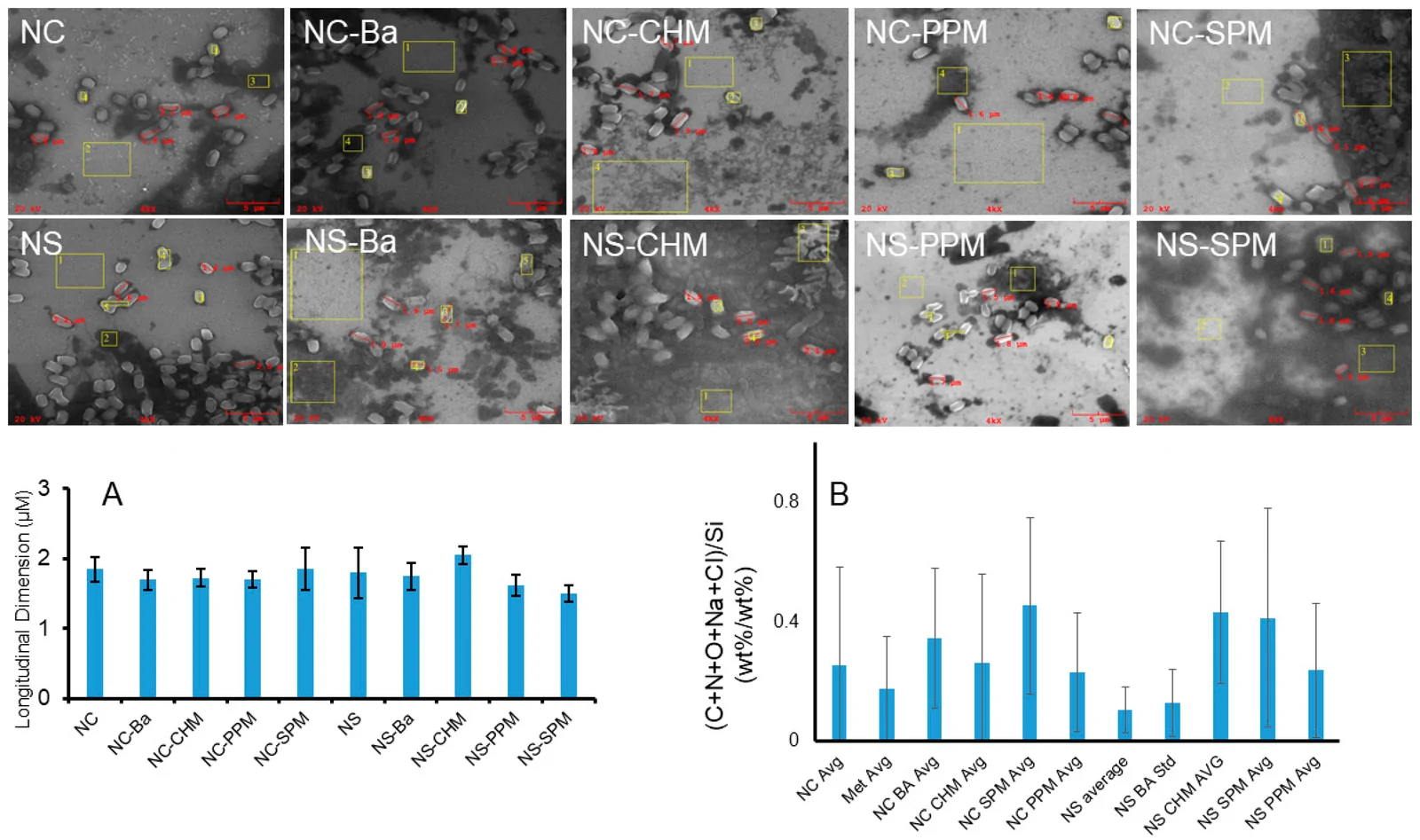
SEM morphology, longitudinal dimensions of dispersed bacterial cells, and four-ROI elemental analysis of *Bacillus thuringiensis* surface-associated material. Representative SEM images show samples formed in chlorine-treated wastewater (NC) and sulfur dioxide-treated wastewater (NS), supplemented with LB medium, under untreated and plant-extract-treated conditions. Red markings indicate the dispersed bacterial cells selected for longitudinal dimension measurements, and yellow boxes indicate the regions of interest (ROIs) selected for EDX analysis. (**A**) Variations in mean longitudinal dimensions of selected bacterial cells in the NC and NS sample series. (**B**) Variations in mean (C + N + O + Na + Cl)/Si ratios calculated from four ROIs for each condition. Error bars represent standard deviations among the measured cells in (**A**) and among the four ROIs in (**B**). The measurements describe variation within the analyzed samples. The corresponding EDX spectra and elemental analyses are provided in Figure S10.

Representative SEM images of *Bacillus thuringiensis (Bt)* biofilms formed on silicon wafers in chlorine-treated wastewater (NC) and sulfur dioxide-treated wastewater (NS), supplemented with LB medium, are shown in Figure 3. The images show the surface morphology of untreated and plant-extract-treated samples. Yellow boxes indicate the regions of interest (ROIs) selected for EDX analysis, and red markings indicate the individual bacterial cells measured for longitudinal dimensions. Differences in surface-associated structures were observed among the experimental conditions. However, the images alone cannot establish changes in total biofilm biomass or bacterial viability.

The longitudinal dimensions of selected dispersed bacterial cells are presented in Figure 3A. In the NC series, the mean dimension was 1.85 ± 0.17 µm for the untreated control. The corresponding values for basil (NC-BA), chocolate mint (NC-CHM), peppermint (NC-PPM), and spearmint (NC-SPM) were 1.70 ± 0.14, 1.73 ± 0.13, 1.70 ± 0.12, and 1.88 ± 0.32 µm, respectively. The mean dimensions for NC-BA, NC-CHM, and NC-PPM were slightly lower than that of the untreated control. NC-SPM showed a similar mean dimension, with greater variation among the measured cells.

In the NS series, the untreated control had a mean longitudinal dimension of 1.80 ± 0.37 µm. The values for NS-BA, NS-CHM, NS-PPM, and NS-SPM were 1.75 ± 0.19, 2.05 ± 0.13, 1.63 ± 0.15, and 1.50 ± 0.12 µm, respectively. NS-CHM had the largest mean dimension among the conditions examined, whereas NS-SPM had the smallest. The mean dimensions of NS-PPM and NS-SPM were lower than that of the untreated NS control. These results show that the measured cell dimensions varied among the plant extracts and wastewater conditions, with no consistent pattern across all treatments. These measurements describe the dimensions of selected dispersed cells and do not, by themselves, establish cell damage, loss of viability, or inhibition of biofilm formation.

EDX analysis was performed on four ROIs for each condition to examine the elemental composition of selected surface regions (Figure 3B and Figure S10). The composite ratio, (C + N + O + Na + Cl)/Si, was calculated for each ROI. The mean and standard deviation were used to describe the variation among the four regions. In the NC series, the ratio was 0.255 for the untreated control and 0.176 for the NC-Methanol control. The corresponding values for NC-BA, NC-CHM, NC-SPM, and NC-PPM were 0.346, 0.262, 0.455, and 0.230, respectively. NC-SPM had the highest mean ratio among the NC conditions, while NC-PPM had the lowest ratio among the plant-extract-treated samples. The NC-Methanol control also showed a lower mean ratio than the untreated NC control. In the NS series, the ratio was 0.104 for the untreated control. The values for NS-BA, NS-CHM, NS-SPM, and NS-PPM were 0.128, 0.432, 0.412, and 0.238, respectively. NS-CHM and NS-SPM had higher mean ratios than the untreated NS control. NS-BA had a ratio close to that of the control, while NS-PPM showed an intermediate value. The differences between the NC and NS series indicate that the relative elemental composition of the analyzed surface regions varied with the wastewater condition and plant-extract treatment.

The standard deviations in Figure 3B were substantial for several conditions, showing that the elemental composition varied among the selected ROIs. These measurements describe local differences across the wafer surface. The composite ratio is a measure of the relative contribution of carbon, nitrogen, oxygen, sodium, and chlorine compared with silicon; it is not a direct measurement of biofilm biomass, extracellular polymeric substances, or bacterial viability. The abiotic controls also showed detectable carbon and nitrogen (Figure S9), indicating that these elements may originate from material deposited from the wastewater–LB medium and plant extracts, as well as from bacterial material. The silicon signal may also be affected by the amount of material covering the underlying wafer.

Taken together, the SEM images, longitudinal dimension measurements, and four-ROI EDX analysis show differences in surface morphology, the dimensions of selected dispersed bacterial cells, and the elemental composition of localized surface regions. The observed patterns varied among the extracts and between the two wastewater conditions.

#### 3.2.3. Biofilm Thickness and Selected Dense-Region EDX Analysis

**Figure 4:**
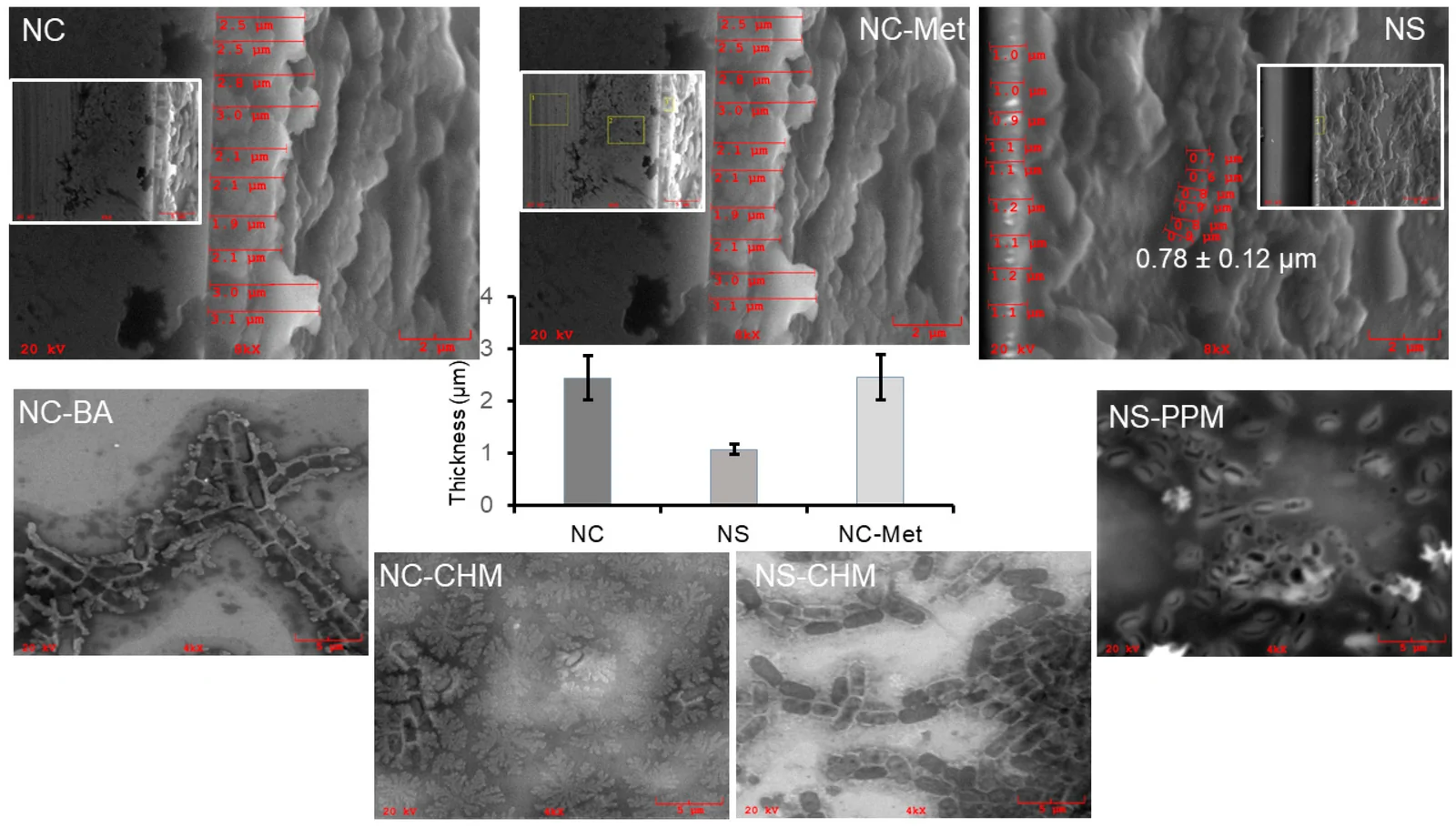
SEM analysis of biofilm thickness and height on silicon wafers. SEM images of biofilms formed under NC, NS, and NC-Met conditions were collected at 8× magnification and a 65° viewing angle to examine their vertical structure and assess biofilm thickness. Red lines indicate the thickness measurements obtained at nine locations for each condition. Mean thickness values (mean ± SD) were 2.44 ± 0.42 µm for NC, 1.08 ± 0.10 µm for NS, and 2.46 ± 0.44 µm for NC-Met which are plotted in the bar graph. The height of the clearly defined biofilm layer in the NS sample was also measured at six locations, and the mean height is presented separately. Additional 4× images are included in the insets for consistency with the plant-extract-treated samples. Representative treated samples (NC-BA, NS-PPM, NC-CHM, and NS-CHM) show scattered, poorly defined surface-associated structures for which reliable thickness measurements could not be obtained. Corresponding EDX spectra and elemental analyses are provided in Figure S11.

SEM images collected at an 8× magnification setting and a 65° viewing angle were used to examine the vertical structure of the NC, NS, and NC-Met samples (Figure 4). The measured mean thickness values were 2.44 ± 0.42 µm for NC, 1.08 ± 0.10 µm for NS, and 2.46 ± 0.44 µm for NC-Met, based on nine measurements per condition and is shown in the bar diagram in Figure 4. The measured thickness for NS was lower than those for NC and NC-Met, whereas the NC-Met value was similar to that of NC. These measurements describe the selected locations in the SEM images and do not represent independent biological replicates.

A separate height measurement was made for the clearly defined biofilm layer in the NS sample. The mean height was 0.78 ± 0.12 µm, based on six measurements. Representative plant-extract-treated samples, including NC-BA, NS-PPM, NC-CHM, and NS-CHM, showed scattered, poorly defined surface-associated structures for which reliable thickness measurements could not be obtained. Therefore, the available images do not support a quantitative thickness comparison between these extract-treated samples and the untreated or methanol-control samples.

To complement the thickness images, EDX analysis was performed on one selected dense region from each condition in a separate sample set. These results are presented in Figure S11. The composite (C + N + O + Na + Cl)/Si ratios were 3.08 for NC, 1.46 for NS, and 3.58 for NC-Met. Lower ratios were measured for the selected regions of NC-BA (0.52), NC-CHM (0.34), NS-CHM (0.76), and NS-PPM (0.76). Thus, the selected dense regions differed in their elemental profiles, with NC-BA and NC-CHM showing lower ratios than the selected NC region. The silicon signal also differed among the selected regions, consistent with differences in the relative contributions of the surface-associated material and underlying substrate to the EDX signal.

The selected dense-region analysis and the four-ROI analysis represent different sampling strategies and separate sample sets. The four-ROI analysis describes spatial variability across sampled locations, whereas the selected dense-region analysis compares the elemental profiles of specifically chosen regions. Because only one dense ROI was analyzed per condition, these ratios are descriptive and do not provide estimates of treatment-wide variability or statistical evidence of differences in biofilm biomass.

Overall, SEM and EDX provide complementary information about the surface-associated structures formed under the different treatment conditions. SEM documents morphology, dispersed-cell dimensions, and measurable vertical structure, while EDX describes the elemental composition of selected regions. The results demonstrate treatment-associated differences in these features, but direct measurements of biofilm biomass and cell viability are needed to determine whether the observed differences reflect changes in biofilm formation.

## 4. Summary and Conclusions

This study combined UPLC–HRMS/MS, SEM, and EDX to characterize four methanolic plant extracts and examine their association with *Bacillus thuringiensis* biofilms formed in treated wastewater supplemented with LB broth. The mass spectrometry analysis showed clear differences in the flavonoid composition of the extracts. Luteolin-7-O-rutinoside was the major quantified flavonoid in peppermint, spearmint, and chocolate mint, whereas basil contained predominantly kaempferol glycosides. The chromatographic separation and MS/MS analysis also provided a means of distinguishing selected flavonoids with identical precursor masses.

The SEM results showed differences in the surface-associated structures among the untreated, methanol-control, and plant-extract-treated samples. The measured longitudinal dimensions of dispersed bacterial cells varied across the experimental conditions, but the changes did not follow a consistent pattern for all extracts. In the representative plant-extract-treated samples examined for vertical structure, the surface-associated material was scattered and poorly defined, so reliable thickness measurements could not be obtained. These observations describe differences in the structures present on the silicon wafers but do not establish changes in bacterial viability or total biofilm biomass.

The EDX analysis provided additional information on the elemental composition of selected surface regions. The four-ROI analysis showed spatial variation within the samples, while the separate analysis of selected dense regions showed differences among the conditions examined in that sample set. The two analyses provide complementary information and should be interpreted separately. The composite elemental ratios are descriptive measurements influenced by material on the wafer surface and the underlying silicon substrate. They cannot independently establish changes in biofilm biomass or extracellular polymeric substance content.

Overall, the results show that the tested plant-extract treatments were associated with differences in the morphology and localized elemental composition of *B. thuringiensis* biofilm-associated structures. The distinct phytochemical profiles provide useful information for future studies of these plant extracts. Further studies on direct measurements of biofilm biomass and bacterial viability are needed to establish the extent of the influence of these extracts on biofilm formation.

## Figures and Tables

**Table 1:** Limit of detection (LOD), quantification (LOQ), and upper limit of quantification (ULOQ) of flavonoids.

Analyte	LOD (ng/mL)	LOQ (ng/mL)	ULOQ (ng/mL)
Fisetin	2.50	5.00	1000
Hesperetin	1.00	5.00	1000
Kaempferol	1.00	5.00	1000
Kaempferol-3-G	1.00	5.00	1000
Kaempferol-3-R	1.00	5.00	1000
Luteolin	1.00	5.00	1000
Luteoloside	1.00	5.00	1000
Luteolin-7-O-R	5.00	7.50	1000
Morin	2.50	5.00	1000
Quercetin	1.00	5.00	1000

**Table 2:** Quantification of selected phytochemicals in peppermint (PPM), spearmint (SPM), chocolate mint (CHM) and basil (BA) extracts. Concentrations are reported as mg mL^−1^ in the original extracts after back-calculation using the applicable sample dilution factor. Values represent means from three analytical replicates, with %CV shown in parentheses.

Sample	Lut7R	Lutd	Lut	Kmp3R	Kmp3G	Quer	Hes
PPM	2.1 × 10^1^ (1.6%)	2.3 × 10^−2^ (0.72%)	2.0 × 10^−4^ (2.3%)	ND	NQ	ND	NQ
SPM	1.9 × 10^1^ (2.3%)	9.7 × 10^−3^ (1.6%)	3.1 × 10^−4^ (2.9%)	ND	NQ	ND	3.4 × 10^−5^ (2.4%)
CHM	2.1 × 10^1^ (2.3%)	3.8 × 10^−3^ (3.6%)	2.8 × 10^−4^ (2.1%)	ND	NQ	ND	5.6 × 10^−5^ (1.8%)
BA	ND	ND	6.3 × 10^−5^ (1.9%)	4.0 × 10^−3^ (3.1%)	9.6 × 10^−4^ (5.8%)	2.7 × 10^−5^ (8.7%)	NQ

Abbreviations: Kaempferol-3-*O*-β-D-glucoside (Kmp3G), kaempferol-3-*O*-rutinoside (Kmp3R), luteolin (Lut), luteoloside (Lutd), luteolin-7-rutinoside (Lut7R), quercetin (Quer), hesperetin (Hes). Values in parentheses are %CV. ND (not detected) and NQ (not quantified) are assigned following Table 1.

## Data Availability

The data presented in this study are available from the corresponding author upon reasonable request.

## Supplementary Materials

The following supporting information can be downloaded at https://www.mdpi.com/article/10.3390/molecules31213836/s1: Figure S1 (UPLC pump gradient for flavonoid analysis, UPLC time program); Table S1 (Physicochemical characterization of NWWTP effluent); Tables S2 and S3 (Q-Exactive Plus full-scan/ddMS^2^ settings, and ddMS^2^ inclusion list); Figures S2–S8 (chromatographic and MS/MS data for isomer differentiation, standards, and flavonoids detected in the plant extracts); Tables S4–S7 (calibration-curve parameters, internal-standard peak-area response, mass calibration, and product-ion qualifiers); Figure S9 (SEM/EDX characterization of abiotic controls); Figure S10 (EDX spectra and corresponding composite elemental ratios for Figure 3); and Figure S11 (selected dense-region EDX spectra and composite elemental ratios corresponding to Figure 4).

## References

[1] World Health Organization 2018: Water, Sanitation, Hygiene, Health: Guidelines on sanitation and health. https://www.who.int/publications/i/item/9789241514705.

[2] UNESCO The United Nations World Water Development Report 2024: Water for Prosperity and Peace. https://unesdoc.unesco.org/ark:/48223/pf0000375724.

[3] Costerton J.W., Stewart P.S., Greenberg E.P. (1999). Bacterial biofilms: A common cause of persistent infections. Science.

[4] Flemming H.-C., Wuertz S. (2019). Bacteria and archaea on Earth and their abundance in biofilms. Nat. Rev. Microbiol..

[5] Flemming H.-C., Wingender J. (2010). The biofilm matrix. Nat. Rev. Microbiol..

[6] Bridier A., Briandet R., Thomas V., Dubois-Brissonnet F. (2011). Resistance of bacterial biofilms to disinfectants: A review. Biofouling.

[7] Sengupta B., Sinha S.S., Garner B.L., Arany I., Corley C., Cobb K., Brown E., Ray P.C. (2016). Influence of Aptamer-Enclosed Silver Nanocluster on the Prevention of Biofilm by *Bacillus thuringiensis*. Nanosci. Nanotechnol. Lett..

[8] Sengupta B., Adhikari P., Mallet E., Havner R., Pradhan P. (2020). Spectroscopic Study on *Pseudomonas aeruginosa* Biofilm in the Presence of the Aptamer-DNA Scaffolded Silver Nanoclusters. Molecules.

[9] Sengupta B., Alrubayan M., Kolla M., Wang Y., Mallet E., Torres A., Solis R., Wang H., Pradhan P. (2025). AI-Based Detection of Optical Microscopic Images of *Pseudomonas aeruginosa* in Planktonic and Biofilm States. Information.

[10] Sengupta B., Ogunlewe O., Friedfeld R., Bess-Grunewald C., Baker P., Onchoke K. (2025). Biofilm-Based Biomonitoring of Treated Wastewater Using *Bacillus thuringiensis*: Toward Sustainable Water Reuse. Sustainability.

[11] Er-rahmani S., Errabiti B., Matencio A., Trotta F., Latrache H., Saad I., El Abed S. (2024). Plant-derived bioactive compounds for the inhibition of biofilm formation: A comprehensive review. Environ. Sci. Pollut. Res..

[12] Borges A., Abreu A.C., Dias C., Saavedra M.J., Borges F., Simões M. (2016). New Perspectives on the Use of Phytochemicals as an Emergent Strategy to Control Bacterial Infections Including Biofilms. Molecules.

[13] Bozin B., Dukic N., Samojlik I., Goran A., Igić R. (2008). Phenolics as antioxidants in garlic, *Allium sativum* L., Alliaceae. Food Chem..

[14] Khameneh B., Iranshahy M., Soheili V., Fazly Bazzaz B.S. (2019). Review on plant antimicrobials: A mechanistic viewpoint. Antimicrob. Resist. Infect. Control.

[15] Silva L.N., Zimmer K.R., Macedo A.J., Trentin D.S. (2016). Plant Natural Products Targeting Bacterial Virulence Factors. Chem. Rev..

[16] AlSheikh H.M.A., Sultan I., Kumar V., Rather I.A., Al-Sheikh H., Tasleem Jan A., Haq Q.M.R. (2020). Plant-Based Phytochemicals as Possible Alternative to Antibiotics in Combating Bacterial Drug Resistance. Antibiotics.

[17] Balakrishnan P., Ramalingam P.S., Suganthi P., Ranganathan B., Gimbun J., Shanmugam K. (2018). A Comprehensive Review on *Ocimum basilicum*. J. Nat. Rem..

[18] Mahendran G., Rahman L.-U. (2020). Ethnomedicinal, phytochemical and pharmacological updates on Peppermint (*Mentha × piperita* L.)—A review. Phytother. Res..

[19] Mimica-Dukic N., Bozin B. (2008). *Mentha* L. species (Lamiaceae) as promising sources of bioactive secondary metabolites. Curr. Pharm. Des..

[20] Royal Horticultural Society (RHS) *Mentha × piperita* f. *citrata *‘Chocolate’. RHS Plant Finder. https://www.rhs.org.uk/plants/104628/mentha-piperita-f-citrata-chocolate/details/.

[21] Schoch C.L., Ciufo S., Domrachev M., Hotton C.L., Kannan S., Khovanskaya R., Leipe D., McVeigh R., O’Neill K., Robbertse B. (2020). NCBI Taxonomy: A comprehensive update on curation, resources and tools. Database.

[22] Sengupta B., Fisher A., Villanueva J., Simington L., Jenkins B., Travis K., Roy D. (2025). Analysis of phytochemicals in watercress leaves using chromatographic, spectroscopic and in-vitro approaches. Results Chem..

