# Supplementary material for: Phytochemical Profiling of Mint and Basil Extracts and Their Association with Wastewater Biofilm-Associated Structures
Source: Molecules. 2026 Oct 23;31(21):3836. doi: 10.3390/molecules31213836 (PMC13764853; doi:10.3390/molecules31213836)
Supplement: Supplementary file 1 [file molecules-31-03836-s001.zip › molecules-4570203-supplementary.pdf]

Phytochemical Profiling of Mint and Basil Extracts and Their Association with Wastewater Biofilm-Associated Structures

[1]Bidisha Sengupta*, [1]Olabisi Ogunlewe, [1]Nkeiruka Angela Aziekwu, [3]Ken Fiffick, [2]Robert Friedfeld, [1]Cephus Bess-Grunewald, [1]Perla Tovar, [1]Philip Baker, [1]Thanh Tien Dao, [3]Kylie Robertson, [3]Michael Janusa, [4]Paul Kim

[1,3]Department of Chemistry and Biochemistry, [2]Department of Engineering and Construction Sciences, Stephen F. Austin State University, 1936 North Street, Nacogdoches, Texas, 75965; [4]Department of Biology, Grambling State University, 403 Main Street, Grambling, Louisiana, 71245

*Corresponding author: Dr. Bidisha Sengupta, email: bidisha.sengupta@sfasu.edu; phone: +1-936-468-2485

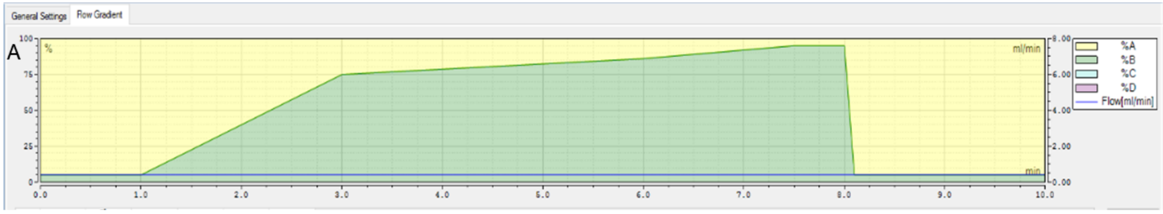

B

Time (min)	Flow (mL/min)	%B
0.000	0.400	5.0
1.000	0.400	5.0
3.000	0.400	75.0
6.000	0.400	86.0
7.500	0.400	95.0
8.000	0.400	95.0
8.100	0.400	5.0
10.000	STOP	STOP

Figure S1. A. Pump gradient for UPLC for Flavonoid Analysis. **B.** Time Program for UPLC for Flavonoid Analysis

Table S1. Physicochemical characteristics of Nacogdoches WWTP effluent during November and December 2024.

Values are means ± standard deviations calculated from daily operational records for each month. n indicates the number of daily records available for the parameter. These records provide operational context and are not direct measurements of the specific wastewater batches used in the biofilm experiments.

Parameter	November 2024 (mean ± SD)	n	December 2024 (mean ± SD)	n
CBOD (mg/L)	2.61 ± 0.71	30	2.89 ± 0.95	31
TSS (mg/L)	3.18 ± 1.62	30	4.25 ± 2.18	31
DO (mg/L)	9.19 ± 0.60	30	9.83 ± 0.67	31
NH_3-N (mg/L)	0.30 ± 0.74	30	0.32 ± 0.59	31
pH	7.22 ± 0.28	30	7.09 ± 0.25	31

Table S2. Q-Exactive Plus settings for Full Scan and ddMS^2 Analysis

Scan Range (m/z)	150.0 to 650.0
Resolution	70,000
Polarity	Negative
Sheath Gas Flow	35
Aux Gas Flow	5
Sweep Gas Flow	1
Spray Voltage (kV)	2.50
Capillary Temperature (°C)	300
Aux Gas Heater Temperature (°C)	275

Table S3. Q-Exactive Plus ddMS^2 Analysis Inclusion List

Analyte	Mass (m/z)	Formula	Species	Polarity	Start (min)	End (min)	CE (V)
Kaempferol	285.04046	$C_{15}H_{10}O_6$	-H	Negative	5.30	5.50	60
Luteolin	285.04046	$C_{15}H_{10}O_6$	-H	Negative	5.11	5.31	55
Fisetin	285.04046	$C_{15}H_{10}O_6$	-H	Negative	4.85	5.05	55
Hesperetin	301.07176	$C_{16}H_{14}O_6$	-H	Negative	5.16	5.36	45
Quercetin	301.03538	$C_{15}H_{10}O_7$	-H	Negative	5.04	5.24	45
Morin	301.03538	$C_{15}H_{10}O_7$	-H	Negative	4.78	4.98	45
Kaempferol-3-G	447.09329	$C_{21}H_{20}O_{11}$	-H	Negative	4.79	4.99	37
Luteoloside	447.09329	$C_{21}H_{20}O_{11}$	-H	Negative	4.51	4.81	37
Kaempferol-3-R	593.15119	$C_{27}H_{30}O_{15}$	-H	Negative	4.77	4.97	35
Luteolin-7-R	593.15119	$C_{27}H_{30}O_{15}$	-H	Negative	4.56	4.76	35

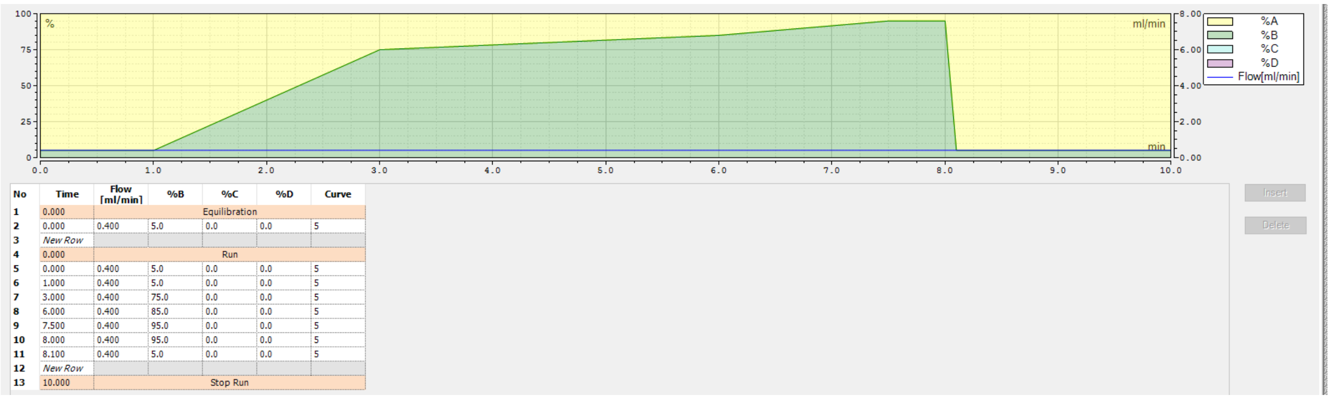

Figure S2. UPLC pump gradient and time program used for chromatographic differentiation of isomeric flavonoid standards. The separation was performed at a flow rate of 0.4 mL min^{-1}. Mobile phase B was maintained at 5% from 0 to 1.0 min, increased linearly to 75% at 3.0 min, followed by an increase to 85% at 6.0 min and 95% at 7.5 min. The 95% B condition was maintained until 8.0 min, after which the composition was returned to 5% B at 8.1 min for column re-equilibration. The total chromatographic run time was 10 min.

Table S4. Calibration curve parameters for the flavonoid panel across multiple batches.

RT (min)	Compound	ISTD	Intercept (×10^{-3})	Slope (×10^{-3})	R^2
Batch 1 Dilution factor: 10					
4.88	Kaempferol-3-R	Fisetin-d5	-1.513	1.200	0.998
4.88	Morin	Fisetin-d5	-6.824	1.752	0.993
4.90	Kaempferol-3-G	Fisetin-d5	-3.305	3.124	0.997
4.96	Fisetin	Fisetin-d5	-37.02	7.147	0.997
5.15	Quercetin	Quercetin-d3	-17.96	8.371	0.998
5.25	Luteolin	Hesperetin-13Cd3	-17.46	7.200	0.996
5.27	Hesperetin	Hesperetin-13Cd3	-9.247	7.322	0.998
5.42	Kaempferol	Hesperetin-13Cd3	-22.75	10.010	0.996
Batch 2 Dilution factor: 1000					
4.70	Luteoloside	Fisetin-d5	48.24	30.440	0.995
4.86	Kaempferol-3-R	Fisetin-d5	5.533	1.503	0.991
4.86	Morin	Fisetin-d5	-4.752	1.168	0.996
4.88	Kaempferol-3-G	Fisetin-d5	13.75	5.477	0.995
4.94	Fisetin	Fisetin-d5	108.7	37.660	1.000
5.13	Quercetin	Quercetin-d3	-7.895	48.270	1.000
5.21	Luteolin	Hesperetin-13Cd3	-142.2	70.030	0.996
5.24	Hesperetin	Hesperetin-13Cd3	-20.75	40.550	1.000
5.38	Kaempferol	Hesperetin-13Cd3	-136.5	76.370	0.997
Batch 3 Dilution factor: 100					
4.7	Luteoloside	Fisetin-d5	48.63	27.660	0.996
4.86	Kaempferol-3-R	Fisetin-d5	1.67	1.235	0.999
4.86	Morin	Fisetin-d5	-2.786	1.087	0.999
4.88	Kaempferol-3-G	Fisetin-d5	5.741	3.898	0.998
4.94	Fisetin	Fisetin-d5	-1.7	35.950	0.999
5.13	Quercetin	Quercetin-d3	-52.74	50.840	1.000
5.21	Luteolin	Hesperetin-13Cd3	-91.68	71.820	0.999
5.24	Hesperetin	Hesperetin-13Cd3	-37.86	39.670	1.000
5.38	Kaempferol	Hesperetin-13Cd3	-119	72.820	0.999
Batch 4 Dilution factor: 100,000					
4.67	Luteolin-7-R	Hesperetin-13Cd3	-0.4001	0.102	0.998

All calibrations used linear regression with 1/x weighting (origin ignored) based on analyte-to-isotope-labeled internal-standard peak-area ratios. RT, retention time.

Table S5. Internal standard peak-area response by batch.

Batch	Internal Standard	Mean area (×10^6)	Area %RSD
1	Fisetin-d5	2.90	7.0
	Quercetin-d3	3.18	8.0
	Hesperetin-13Cd3	7.51	11.4
2	Fisetin-d5	4.64	7.6
	Quercetin-d3	3.39	16.8
	Hesperetin-13Cd3	5.12	14.1
3	Fisetin-d5	5.50	8.3
	Quercetin-d3	3.68	9.9
	Hesperetin-13Cd3	5.30	14.1
4	Hesperetin-13Cd3	6.14	2.1

%RSD is calculated across all injections in a batch.

Table S6. Mass calibration of the Q-Exactive Plus performed prior to each batch

Batch	RMS mass error (ppm)
1	0.28 / 0.35
2	0.28 / 0.29
3	0.30 / 0.31
4	0.26 / 0.28

RMS is root-mean-squared mass error across calibrant ions

Table S7. Product ion qualifiers for each ddms^2 experiment. Top three most abundant product ions were selected.

Compound	Product Ion Qualifier 1 (m/z)	Product Ion Qualifier 2 (m/z)	Product Ion Qualifier 3 (m/z)
Fisetin	121.0282	135.0074	91.0176
Luteolin	133.0282	151.0024	107.0125
Kaempferol	133.0282	93.0332	178.9915
Quercetin	151.0025	121.0283	107.0126
Luteolin-7-O-R	285.0398	286.0433	157.0053
Kaempferol-3-R	285.0406	284.0329	255.0298
Luteoloside	285.0396	284.0323	286.043
Kaemperol-3-G	284.0327	285.0386	255.0299
Hesperetin	164.0104	151.0025	196.0004

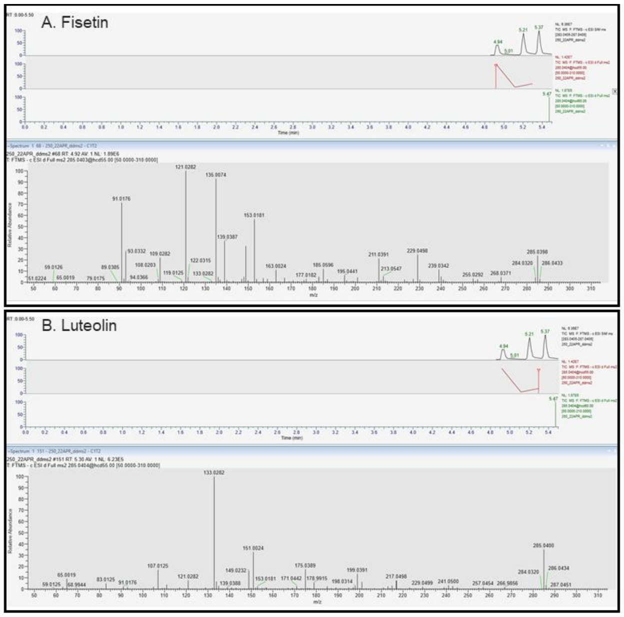

Figure S3. Representative negative-mode ddMS² spectra of fisetin (A) and luteolin (B), showing distinct product-ion profiles despite their identical precursor mass (m/z 285.040).

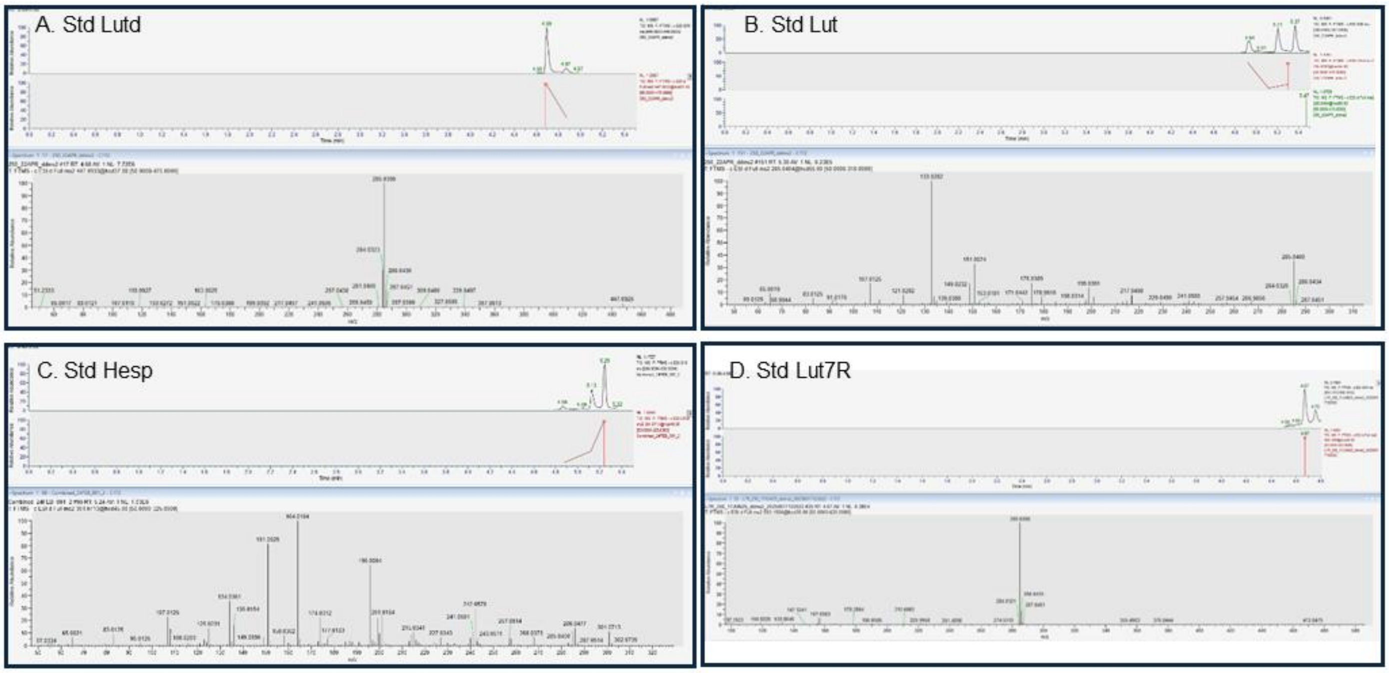

Figure S4. Representative UPLC– HRMS ddMS² chromatograms and corresponding MS/MS spectra of selected flavonoid standards (std): A, B, C, and D correspond to Luteoloside (Lutd), luteolin (Lut), hesperetin (Hesp), and luteolin-7-O-rutinoside (Lut7R), respectively which are detected in mint extracts.

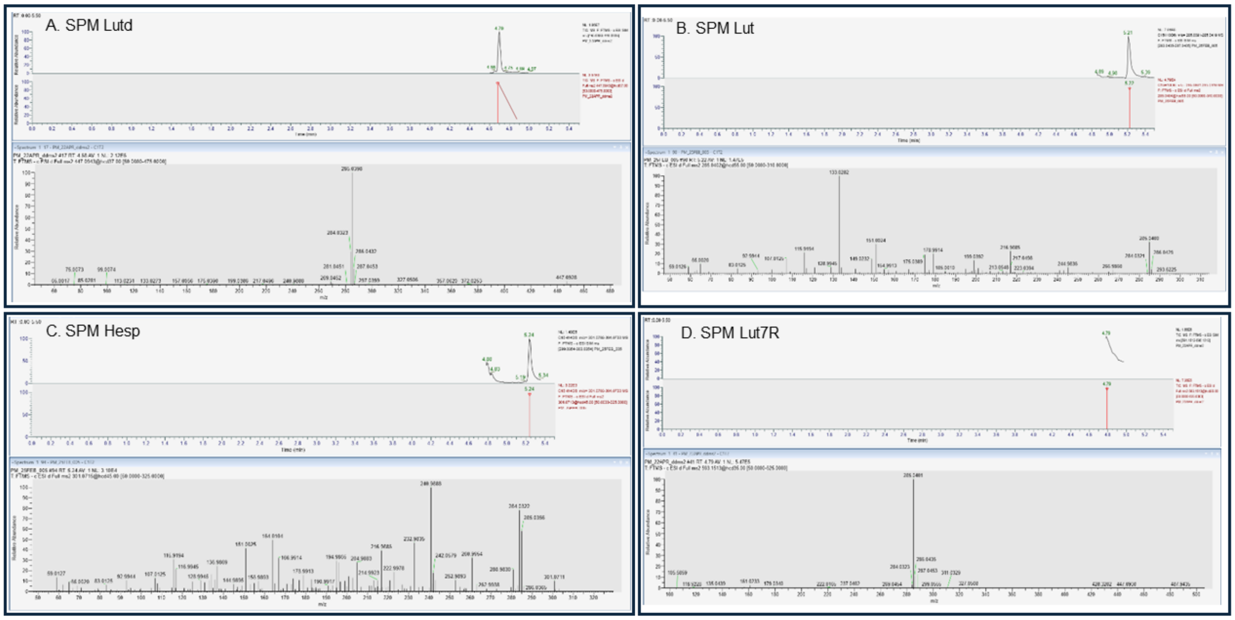

Figure S5. Representative UPLC–HRMS ddMS² chromatograms and corresponding MS/MS spectra of selected flavonoids in spearmint (SPM) extract. A, B, C, and D correspond to Luteoloside (Lutd), luteolin (Lut), hesperetin (Hesp), and luteolin-7-O-rutinoside (Lut7R), respectively. Compound assignments were based on comparison of chromatographic retention times, accurate precursor masses, and MS/MSfragmentation characteristics between the spearmint extracts and authentic standards (Figure S4).

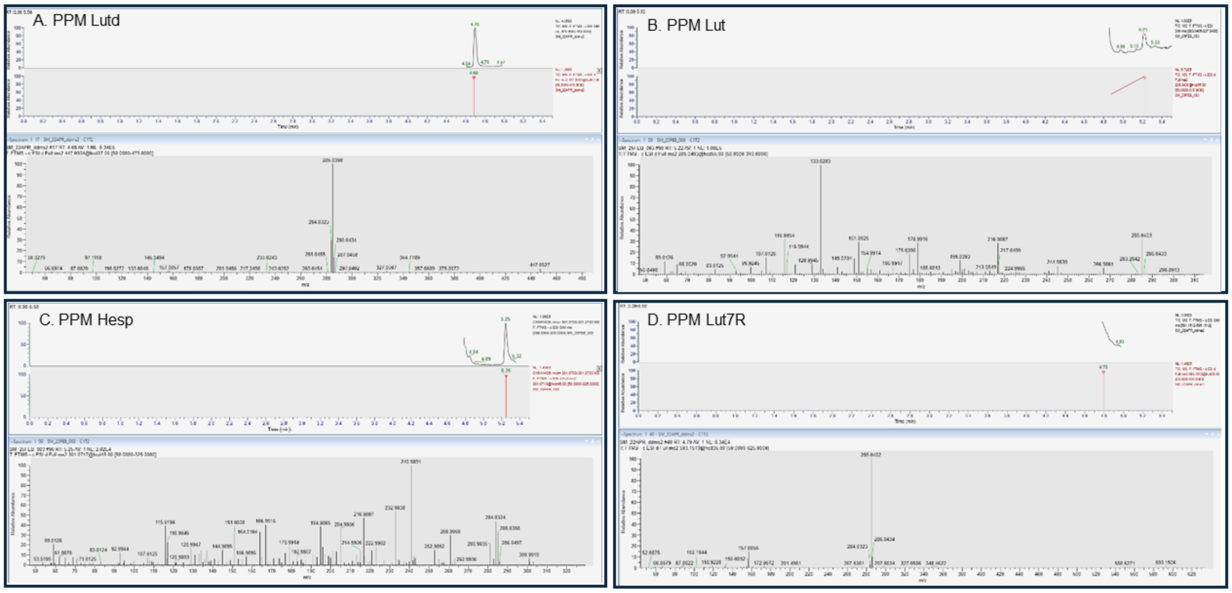

Figure S6. Representative UPLC–HRMS ddMS² chromatograms and corresponding MS/MS spectra of selected flavonoids in peppermint extract (PPM). (A) Luteoloside (Lutd) detected in peppermint extract and (B) luteolin detected in peppermint extract (Lut); (C) hesperetin (Hesp) detected in peppermint extract; and (D) luteolin-7-O-rutinoside (Lut7R) detected in peppermint extract. Agreement in chromatographic retention times and characteristic precursor/product-ion patterns between the extract and corresponding authentic standards (Figure S4) supports the identification of these flavonoids in peppermint.

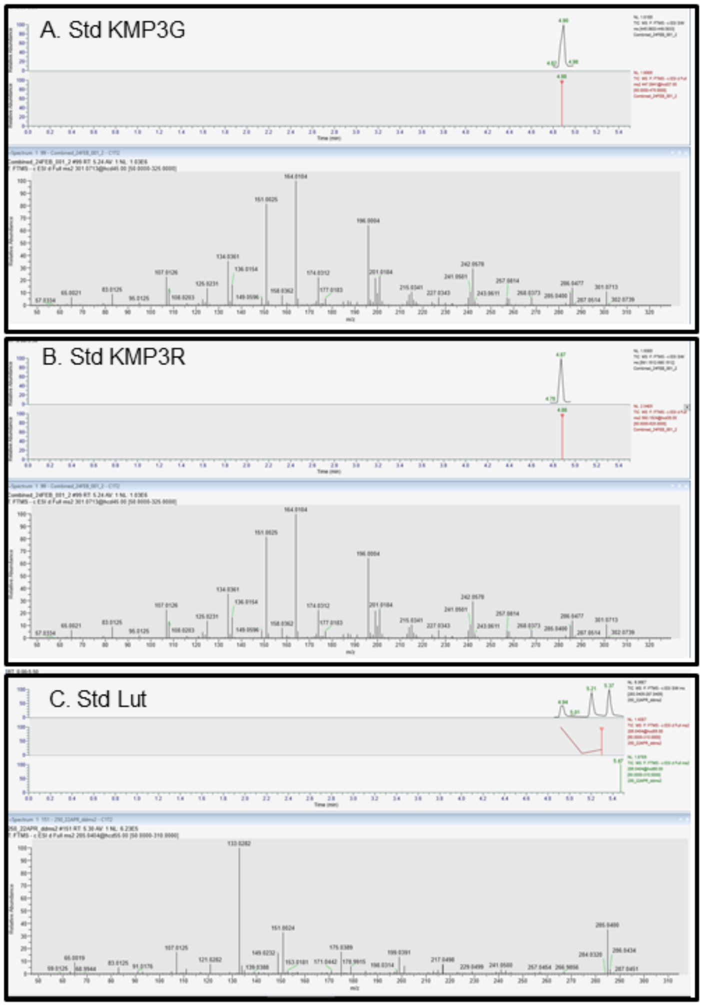

Figure S7. Representative UPLC–HRMS ddMS² chromatograms and corresponding MS/MS spectra of selected flavonoids standards (std) (A) Kaempferol-3-O-glucoside (KMP3G); (B) kaempferol-3-O-rutinoside (KMP3R); (C) luteolin (Lut) as detected in basil extract.

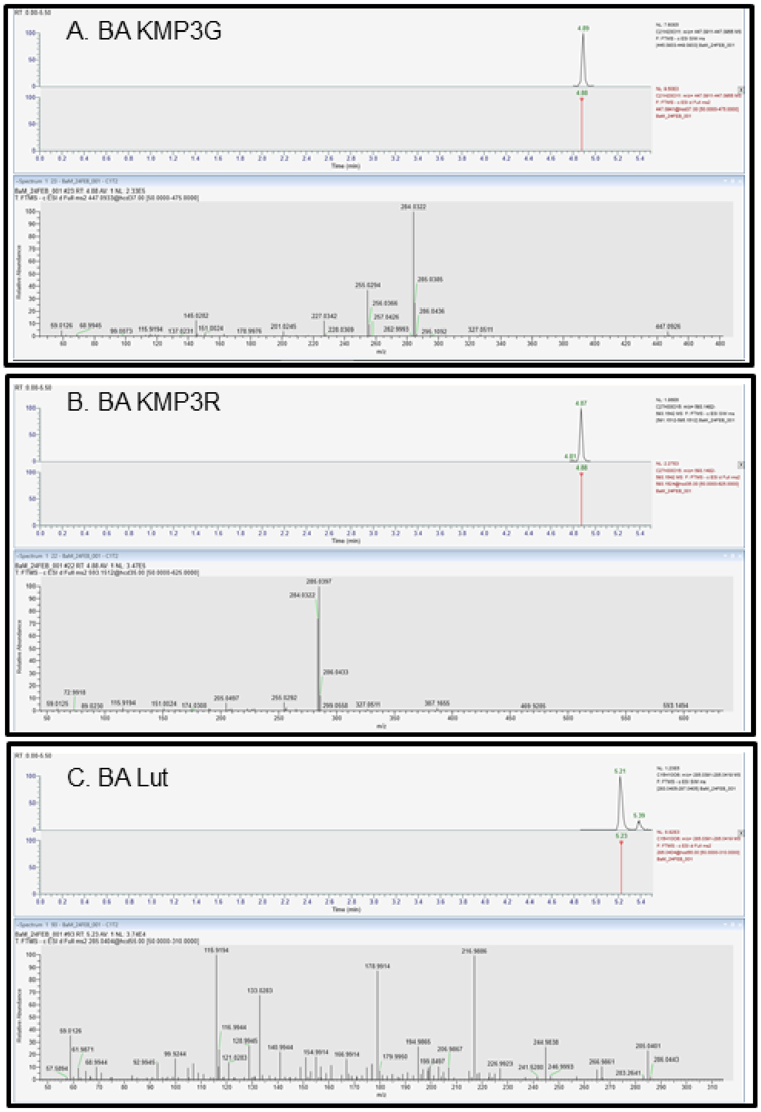

Figure S8. Representative UPLC–HRMS ddMS^2 chromatograms and corresponding MS/MS spectra of selected flavonoids detected in basil extract (BA). (A) Kaempferol-3-O-glucoside (KMP3G) detected in basil extract and (B) kaempferol-3-O-rutinoside (KMP3R) detected in basil extract; and (C) luteolin (Lut) detected in basil extract. The agreement in chromatographic retention times and characteristic precursor/product-ion patterns between the basil extract and corresponding authentic standards (Figure S7) supports the assignment of these flavonoids in basil.

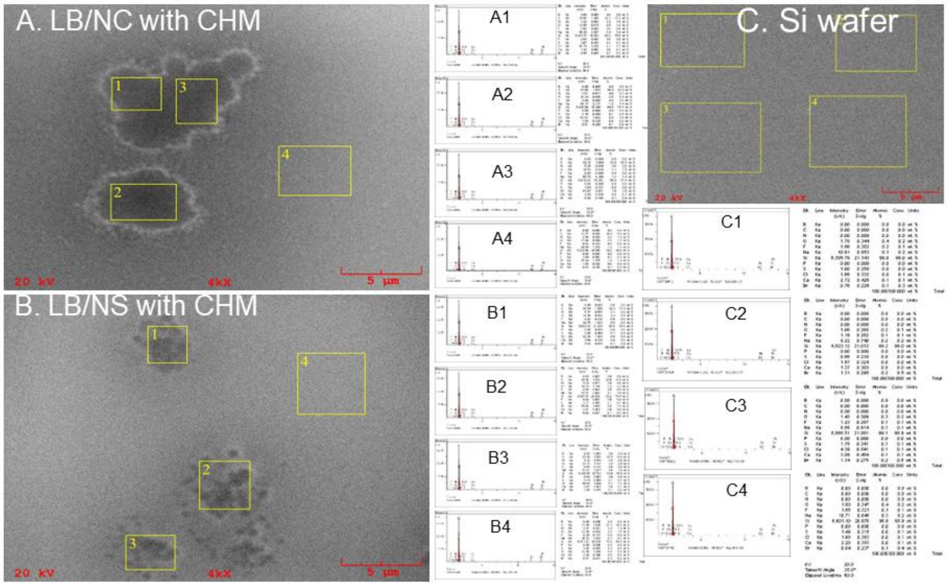

Mean elemental composition (wt.%) of the Si wafer covered with abiotic environments. A: 50% LB/NC with 0.3% CHM; B: 50% LB/NS with 0.3% CHM; and C: blank, as determined by EDX analysis (n = 4 ROIs).

Elements	Line	Si wafer	LB/NC/CHM	LB/NS/CHM
C	Ka	0.0	16.3	14.0
N	Ka	0.0	2.2	1.9
O	Ka	0.2	1.7	2.0
F	Ka	0.1	0.0	0.0
Na	Ka	0.2	1.3	0.9
Si	Ka	99.0	76.5	79.9
P	Ka	0.0	0.0	0.0
S	Ka	0.1	0.1	0.1
Cl	Ka	0.1	1.6	1.0
Br	Ka	0.4	0.3	0.3
Total		100.0	100.0	100.0

Figure S9. SEM images (A, B and C) and EDX analysis (A1-A4, B1-B4, and C1-C4) of abiotic controls on silicon wafers. (A) Silicon wafer exposed to the abiotic LB/NC matrix (50% v/v) containing 0.3% chocolate mint (CHM) extract. (B) Silicon wafer exposed to the abiotic LB/NS matrix (50% v/v) containing 0.3% CHM extract. (C) Unexposed silicon wafer. Yellow boxes indicate the four regions of interest (ROIs) analyzed by EDX. The table summarizes the mean elemental composition (wt%) across four ROIs for each condition. The presence of carbon and nitrogen in the abiotic controls indicates that these elements may originate from the treatment matrix as well as from biological material in the biofilm experiments.

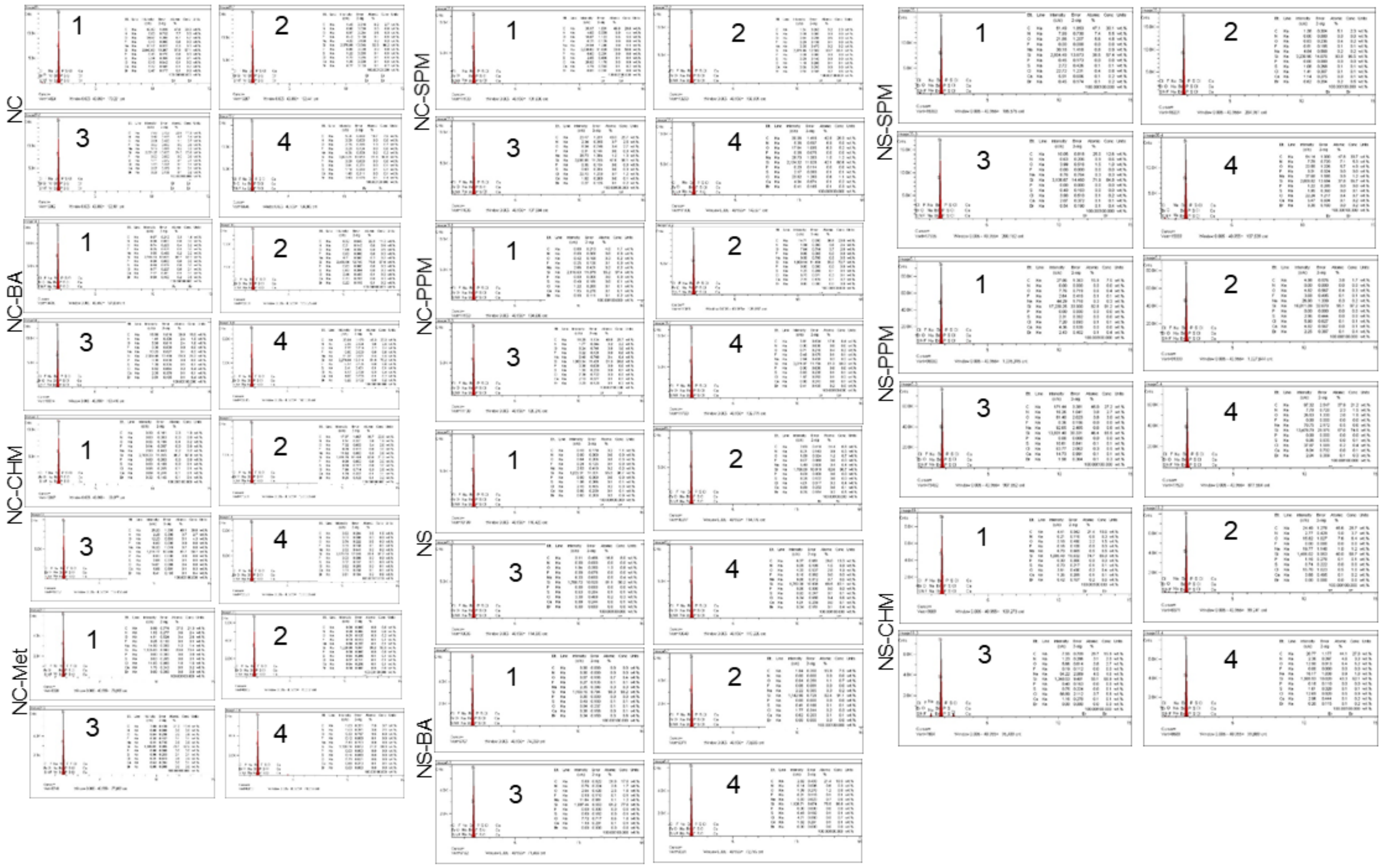

Figure S10. EDX spectra and corresponding elemental composition analyses for four regions of interest (ROIs) per sample condition corresponding to Figure 3. EDX spectra and their corresponding elemental composition tables (insets) are presented for ROIs 1–4 for each indicated condition. Samples include the control conditions (NC and NS), methanol control (NC-Met), and plant-extract treatments (BA, CHM, PPM, and SPM) under the chlorine-treated wastewater (NC) and sulfur dioxide-treated wastewater (NS) conditions, as indicated by the sample labels. The four ROIs characterize spatial variation in elemental composition across the analyzed surface regions. These ROIs represent spatial subsampling and should not be interpreted as independent biological replicates.

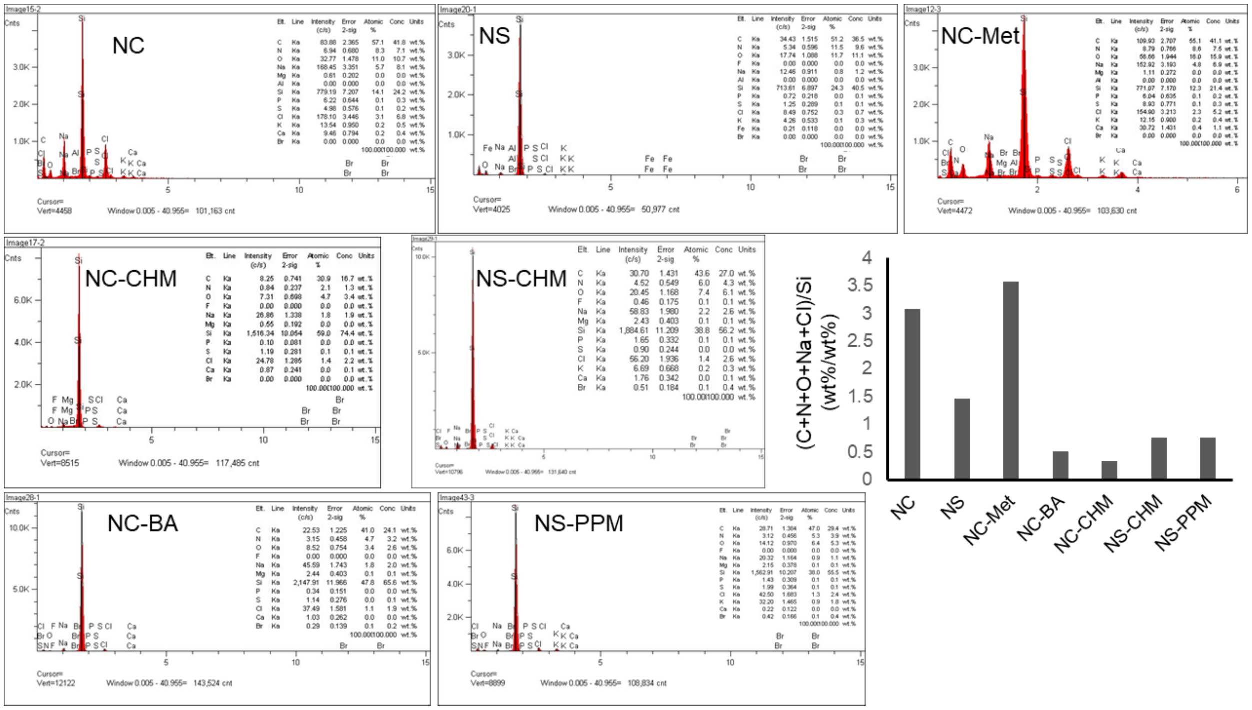

Figure S11. Representative EDX spectra are shown for NC, NS, NC-Met, NC-CHM, NS-CHM, NC-BA, and NS-PPM. The bar graph compares the combined weight ratio of carbon, nitrogen, oxygen, sodium, and chlorine relative to silicon, expressed as ((C+N+O+Na+Cl)/Si). EDX analysis was performed on a selected dense region for each condition. Differences in the ratios indicate variation in the elemental composition of the selected biofilm-associated regions among the treatment conditions.
